# Identification of Indigenous Thai *Phlegmariurus* Genotypic Population by Integrating Morphological and Molecular Studies

**DOI:** 10.3390/plants14091400

**Published:** 2025-05-07

**Authors:** Nusanisa Chedao, Avinash Chandra Pandey, Potjamarn Suraninpong

**Affiliations:** 1 Faculty of Agriculture, Princess of Naradhiwas University, Narathiwat 96000, Thailand; nusanisa.c@pnu.ac.th; 2 Center of Excellence on Agricultural Biotechnology: (AG-BIO/MHESI), Bangkok 10900, Thailand; 3 School of Agricultural Technology and Food Industry, Walailak University, Nakhon Si Thammarat 80161, Thailand; 4 Barley R&D Division, Barmalt Malting India Private Limited, Kotputli 303108, Rajasthan, India; yuvrajavi@gmail.com; 5 Herbology Research Center, Walailak University, Nakhon Si Thammarat 80160, Thailand

**Keywords:** lycopodiceae, *Phlegmariurus*, AFLP, SSR, *rbcL*, *psbA-trnH*

## Abstract

*Phlegmariurus*, a diverse genus within the Lycopodiaceae family, has wide diversity in tropical regions, including Thailand. Accurate species delimitation in the tropical clubmoss genus *Phlegmariurus* is challenged by high morphological plasticity and genetic complexity. This study applied an integrative multilocus approach combining morphometric analysis of 27 complete specimens, 35 *Phlegmariurus* and *one Lycopodiella* accessions for AFLP genotyping (926 loci; PIC 0.32), SSR profiling (44 loci; PIC 0.57; expected heterozygosity 0.35), and chloroplast barcoding using *rbcL* (1308 bp; bootstrap 89–99%) and the *psbA*-*trnH* intergenic spacer (308 bp; bootstrap ≥ 94%). A total of 13 were identified as belonging to seven known species, including *P. nummulariifolius* (NST01, NST15, NST36), *P. goebelii* (JP04), *P. phlegmaria* (NST13), *P. verticillatus* (PHI16), *P. squarrosus* (NST21, NST22, MY31), *P. tetrastichus* (NST30), and *P. carinatus* (MY32, MY33, NST34). Morphological clustering and molecular markers consistently distinguished *Phlegmariurus* accessions from the *Lycopodiella* outgroup. Additionally, 19 previously unclassified *Phlegmariurus* accessions were successfully identified as belonging to the species *P. nummulariifolius* (NST23), *P. goebelii* (NST03, JP05, STN12, PNA14, SKA25, CPN26, KRB27, PNA28), *P. phlegmaria* (NWT07, STN08, NST09, NST10, PHI29), *P. squarrosus* (NST17), and *P. carinatus* (PNA06, STN18, CPN19, JP24). Moreover, this study identified three novel lineages (NST02, STN11, NST20) with strong support across datasets. The combination of broad genomic coverage (AFLP), fine-scale allelic resolution (SSR), deep-branch backbone (*rbcL*), and terminal-branch discrimination (*psb*A-*trn*H) yields a robust framework for species identification. These results define clear operational units for conservation prioritization and establish a foundation for marker-assisted development of ornamental *Phlegmariurus* cultivars.

## 1. Introduction

The family Lycopodiaceae (class Lycopodiopsida, order Lycopodiales) comprises approximately 17 accepted genera and an estimated 400–500 species worldwide. Based on molecular evidence, the family is classified into three subfamilies—Lycopodielloideae, Lycopodioideae, and Huperzioideae [1]. A morphometric analysis in Southeast Asia further divided the family into three genera: *Huperzia*, *Lycopodiella*, and *Lycopodium* s. str [2]. *Huperzia* and *Lycopodium* exhibited more variation in traits, while *Lycopodiella* was more homogeneous [2,3]. In Southeast Asia, there are 25 species of *Huperzia*, 2 species of *Lycopodiella*, and 8 species of *Lycopodium*, with Thailand hosting 13 species of *Huperzia* [4,5].

The taxonomic history of *Phlegmariurus* has undergone significant revisions [1]. Initially classified within *Huperzia* due to morphological similarities [3,6,7,8], molecular phylogenetic studies later revealed that *Huperzia* s.l. was paraphyletic with respect to *Phylloglossum* [9,10]. *Phlegmariurus* was eventually recognized as a distinct genus within the Huperziaceae family, which led to the proposal to split *Huperzia* into two genera [11,12]. *Huperzia* was previously divided into two sections based on leaf morphology: section *Huperzia* (entire leaves) and section *Serratae* (serrate or denticulate leaves) [13,14]. In China, *Phlegmariurus* was further classified into three sections: section *Huperzioides* H. S. Kung and L. B. Zhang, section *Carinaturus* (Herter) H. S. Kung and L. B. Zhang, and section *Phlegmariurus* [15,16].

A key distinction between *Huperzia* and *Phlegmariurus* lies in their reproductive structures and spore morphology. *Huperzia* produces gemmae—asexual reproductive structures that detach and develop into new individuals—while *Phlegmariurus* does not [1]. Although this distinction is recognized in the Flora of North America [17], *Phlegmariurus* remains difficult to separate morphologically, leading some taxonomists to support a one-genus classification for the subfamily [10]. Under the revised classification, as of June 2024, the Checklist of Ferns and Lycophytes of the World accepted over 300 species of *Phlegmariurus*, while *Huperzia* contains around 25 species [18].

*Phlegmariurus* exhibits remarkable morphological and ecological diversity, occurring from sea level to elevations of 5000 m in habitats ranging from montane forests to alpine grasslands. It adapts to various growth forms—epiphytic, terrestrial, and rupicolous—resulting in significant variation in growth habit, leaf morphology, phyllotaxy, and fertile-sterile leaf dimorphy [10]. Modern taxonomic frameworks continue to refine evolutionary relationships within the family. However, the classification of Lycopodiaceae remains complex, with frequent reclassifications contributing to uncertainty in generic delineation.

Molecular markers have significantly advanced studies of genetic variation, species identification, and quantitative trait locus (QTL) discovery for marker-assisted selection. AFLP is a robust DNA fingerprinting technique capable of detecting genetic variation across multiple loci without requiring prior sequence information [19,20]. Similarly, SSR markers are widely applied in genetic diversity analysis, molecular breeding, and germplasm conservation [21]. Although both AFLP and SSR are highly reproducible and effective in detecting polymorphisms, SSR markers differ in that they require prior knowledge of flanking DNA sequences for locus-specific primer design—a process that is often costly and dependent on next-generation sequencing (NGS) technologies. Despite this, SSR markers remain valuable tools for DNA fingerprinting and population diversity analysis. In *H. serrata*, for example, microsatellite markers have been developed to assess the species’ evolutionary potential [22].

DNA barcoding provides a rapid and accurate method for species identification, which is crucial for ecological studies [23]. Chloroplast DNA (cpDNA) plays a key role in genetic research, with regions such as *psbA-trnH* aiding in species identification, despite challenges like sequence inversions [24]. The *rbcL* gene is widely used in phylogenetic analyses, although *Phlegmariurus* appears to be polyphyletic, and there is weak support for the monophyly of the Lycopodiaceae family [25]. Phylogenetic analysis of Phlegmariuraceae using *rbcL* and *psbA-trnH* revealed two major clades, suggesting a complex evolutionary history within the genus and identifying sister group relationships among Chinese *Phlegmariurus* species [26]. Chloroplast rDNA has also been used to study Lycopodiaceae, supporting *Lycopodium* and *Lycopodiella* as sister taxa and suggesting that *H. selago* should be recognized as a separate genus [27]. This study integrates morphological characteristics, AFLP, SSR markers, and chloroplast sequence data (*rbcL* and *psbA-trnH*) to refine the phylogenetic understanding of *Phlegmariurus* species in Southern Thailand.

## 2. Results

### 2.1. Morphology and Cluster Analysis of Phlegmariurus Within Southern Thailand

#### 2.1.1. Morphology Study

Morphometric analysis of 27 *Phlegmariurus* samples and one *Lycopodiella* species, based on five qualitative and ten quantitative traits, revealed clear distinctions between these genera in Southern Thailand. *Phlegmariurus*, an epiphytic tassel fern, exhibited larger, more robust structures, while *Lycopodiella*, a terrestrial clubmoss, had a more slender and creeping growth form. Quantitative comparisons between *Phlegmariurus* and *Lycopodiella* further supported the morphological distinctions. *Phlegmariurus* exhibited a significantly larger main stem diameter (4.17 ± 2.40 cm) than *Lycopodiella* (0.60 ± 0.00 cm), as well as longer fertile branches and strobili. Microphylls of *Phlegmariurus* were both longer (1.00 ± 0.55 cm) and slightly wider (0.40 ± 0.22 cm) than those of *Lycopodiella*. The distance from the apex to the widest portion of the microphyll was also greater in *Phlegmariurus* (0.44 ± 0.22 mm). In contrast, *Lycopodiella* had longer sporophylls (1.37 ± 0.00 mm) compared to *Phlegmariurus* (0.43 ± 0.31 mm), despite the latter having wider strobili and sporophylls. These differences across the ten measured traits underscore the distinct vegetative and reproductive morphologies of the two genera (Table 1).

Qualitative traits further distinguished the two genera. Most *Phlegmariurus* leaves were angled at 45°, but some samples (NST09, NST10, STN11, NST13) had a 90° orientation. Leaf apex variation included mucronate forms in NST02 and STN11, while others were acute. Leaf shape ranged from ovate to lanceolate or elliptic, with NST15 uniquely elliptic. Leaf bases varied across acute, attenuate, obtuse, and truncate forms. These traits provide key diagnostic features for distinguishing the two genera (Supplementary Table S1).

#### 2.1.2. Principal Component Analysis (PCA) of Morphological Clustering in *Phlegmariurus* Specimens

PCA findings based on 15 morphological traits revealed distinct patterns of variation among 27 *Phlegmairurus* specimens. The first principal component (PC1) accounted for 87.57% of the total variance, while the second principal component (PC2) accounted for an additional 2.30%. The highest loading on PC1 was observed for the trait distance from apex to the widest portion of the microphyll (loading = 0.992). All other traits contributed minimally to PC1, with loadings below |0.1|. In comparison, PC2 explained only 2.3% of the variance, with higher loadings from leaf apex (−0.56), length of fertile branches (−0.54), leaf shape (−0.324), and sporophyll width (−0.289). These traits showed relatively higher influence on PC2 compared to others (Table 2).

#### 2.1.3. PCA Biplot and Interpretation

**Figure 1:**
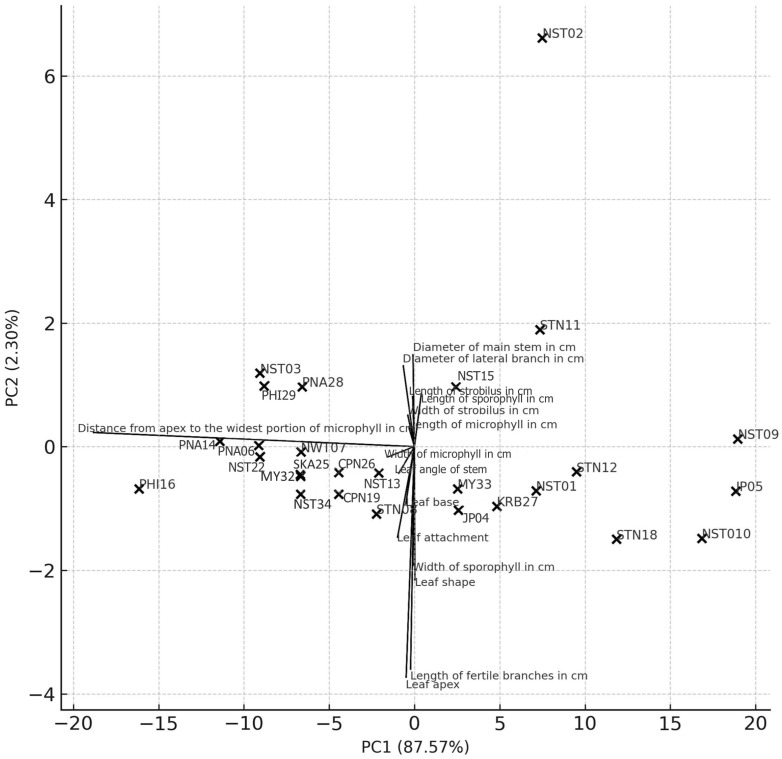
PCA biplot of 27 *Phlegmariurus* specimens based on 15 morphological traits, including 10 quantitative and 5 scored qualitative characters. PCA biplot with trait loading vectors (line), illustrating the relative contribution and directionality of each morphological trait in shaping the principal component space.

The PCA biplot with variable loadings illustrates the distribution of 27 *Phlegmariurus* specimens in relation to the first two principal components, which together explain 89.87% of the total morphological variation (PC1: 87.57%, PC2: 2.30%). Specimens could be separated into three main groupings. Specimens on the far left of the plot (PC1 < −5) include PHI16, PNA14, PNA06, NST03, PHI29, NST22, NWT07, SKA25, MY32, PNA28, and NST34. These individuals are characterized by smaller values for key vegetative traits such as stem diameter and leaf size. Specimens clustered near the center (PC1 between −5 and +5) include STN08, JP04, MY33, NST13, CPN26, CPN19, KRB27, and NST15. These specimens exhibit intermediate values for most traits and reflect relatively homogeneous morphological features. Specimens on the right side of the plot (PC1 > +5) include NST01, NST02, NST09, JP05, NST10, STN11, STN12, and STN18. This group is strongly influenced by positive loadings on vegetative variables such as stem diameter, strobilus size, and microphyll width. Notably, NST02 is distinctly positioned away from the other specimens along PC2, reflecting a unique combination of traits including increased strobilus size and stem thickness. The directions and lengths of trait vectors indicate that PC1 is dominated by variables related to plant robustness (e.g., diameter of main stem, width of microphyll, and strobilus size), while PC2 contributes to minor variation associated with traits such as leaf shape, length of fertile branches, and angle of strobilus (Figure 1).

#### 2.1.4. Hierarchical Clustering Analysis

Hierarchical cluster analysis separated the 27 *Phlegmariurus* specimens into two primary clusters, designated as Cluster A and Cluster B. These two main clusters were further subdivided into a total of six morphologically distinct subgroups, reflecting structured patterns of variation among the specimens.

Cluster A comprised two subgroups. Subgroup 1A consisted of nine specimens: STN08, NST13, NWT07, PNA28, CPN19, CPN26, NST34, SKA25, and MY32, which appeared morphologically similar and moderately divergent from the remaining samples. Subgroup 2A included PHI16, NST03, PNA14, PHI29, PNA06, and NST22, which shared another distinct morphological profile.

**Figure 2:**
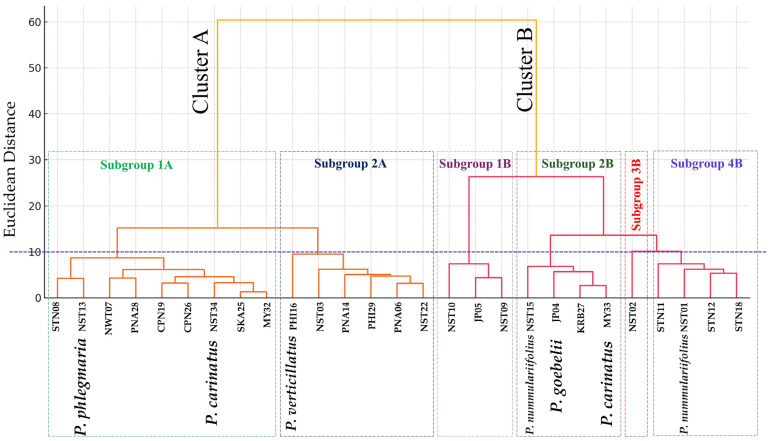
Hierarchical clustering dendrogram of 27 *Phlegmariurus* specimens based on 15 standardized morphological traits using Ward’s method and Euclidean distance. Color-coded branches indicate subgroup memberships, consistent with PCA results. Specimen codes match those used in PCA and morphometric analyses. Specimens without species names represent unidentified *Phlegmariurus* and were included to assess intra-and interspecific morphological variation.

Cluster B exhibited greater heterogeneity, comprising four subgroups. Subgroup 1B contained three specimens—NST10, JP05, and NST09—characterized by relatively robust vegetative features. Subgroup 2B included NST15, JP04, KRB27, and MY33, representing specimens with moderate morphological traits. In contrast, Subgroup 3B was represented by a single specimen, NST02, which displayed unique morphological characteristics, as also seen in the PCA biplot. Lastly, Subgroup 4B consisted of STN11, NST01, STN12, and STN18, which formed a separate cluster with a shared morphotype (Figure 2).

### 2.2. Analysis of Genetic Variability Among Phlegmariurus Species Using AFLP and SSR Marker

#### 2.2.1. Amplified Fragment Length Polymorphism Analysis (AFLP)

AFLP analysis using ten primer combinations yielded a total of 974 scorable loci across 36 specimens and 95.07% of which were polymorphic. Fragment sizes ranged from 100 to 400 bp, with each primer combination producing an average of 92.6 polymorphic loci (Supplementary Figure S1). The number of polymorphic fragments per primer varied from 69 (E-AT/M-CGA) to 129 (E-AG/M-CGA). Jaccard’s genetic similarity coefficients ranged from 0.410 to 0.818, with the lowest similarity (0.410) observed between MY31 and LYCO35 (Supplementary Table S2). Polymorphism Information Content (PIC) values ranged from 0.21 to 0.50, averaging 0.32. Notably, seven loci (0.72%) reached the maximum PIC value of 0.50, while 336 loci (34.5%) exceeded a PIC of 0.30 (Table 3).

**Figure 3:**
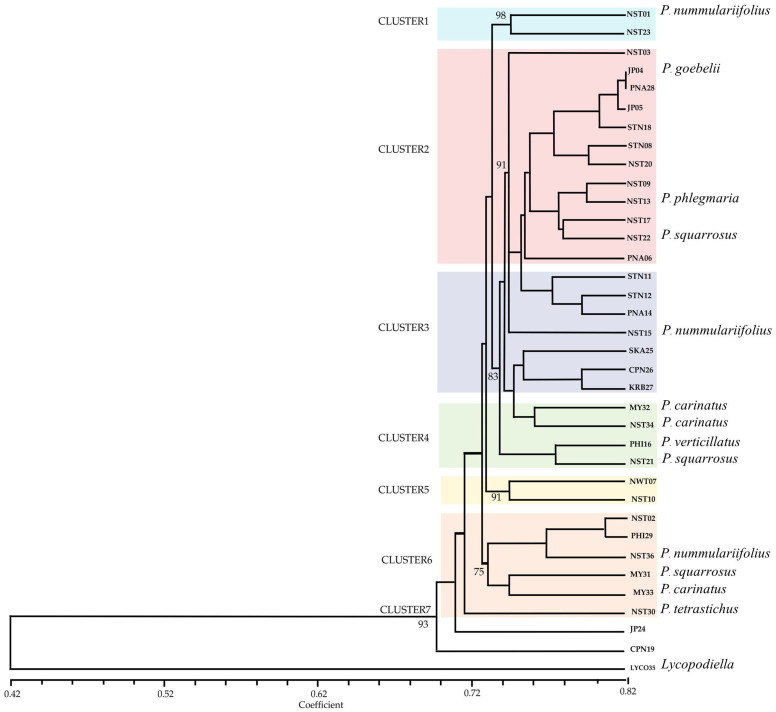
UPGMA dendrogram showing the genetic relationships among 35 *Phlegmariurus* and *Lycopodiella* specimens based on AFLP markers. Genetic similarity was calculated using Jaccard’s coefficient, and clustering was performed using the unweighted pair group method with arithmetic mean (UPGMA). The horizontal axis represents the Jaccard similarity coefficient, ranging from 0.48 to 0.85. Bootstrap values (1000 permutations) are shown at each node to indicate branch support. LYCO35 (*Lycopodiella*) was included as an outgroup. Specimens without assigned species names represent *Phlegmariurus* accessions included to assess intra-generic genetic variation.

Hierarchical clustering analysis based on AFLP markers and Jaccard similarity coefficients grouped 36 specimens into seven distinct genetic clusters (Figure 3). The clustering was performed using the UPMGA method, with branch distances ranging from 0.42 to 0.82.

Cluster 1 included NST01 (*P. nummularifolius*) and NST23, which exhibited a high pairwise Jaccard similarity in the dataset (0.770).

Cluster 2 comprised NST03, JP04 (*P. goebelii*), PNA28, JP05, STN18, STN08, NST20, NST09, NST13 (*P. phlegmaria*), NST17, NST22 (*P. squarrosus*), and PNA06. Pairwise Jaccard similarities within this cluster ranged from 0.756 to 0.818. Within this cluster, several pairs, such as NST13 and NST17 (0.785), also showed high similarity.

Cluster 3 grouped STN11, STN12, PNA14, NST15 (*P. nummularifolius*), SKA25, CPN26, and KRB27. The pairwise similarity between NST15 and PNA14 was 0.797.

Cluster 4 contained MY32 (*P. carinatus*), NST34 (*P. carinatus*), PHI16 (*P. verticillatus*), and NST21 (*P. squarrosus*), where similarities fell within the range of 0.761 to 0.788, representing closely related genotypes with some interspecific overlap.

Cluster 5 included NWT07 and NST10, which formed a small yet genetically distinct branch (coefficient ~0.753).

Cluster 6 contained NST02, PHI29, NST36 (*P. nummularifolius*), MY31 (*P. squarrosus*), MY33 (*P. carinatus*), and NST30 (*P. tetrastichus*). Despite shared clustering, genetic similarity among these samples was more variable (range from 0.747 to 0.783).

Cluster 7 included JP24 and CPN19, while the outgroup is LYCO35 (*Lycopodiella* sp.), was genetically distinct and clustered separately. The lowest similarity observed in the matrix was 0.410 between MY31 and LYCO35, confirming a strong genetic divergence between the genera *Phlegmariurus* and *Lycopodiella* (Figure 3).

**Figure 4:**
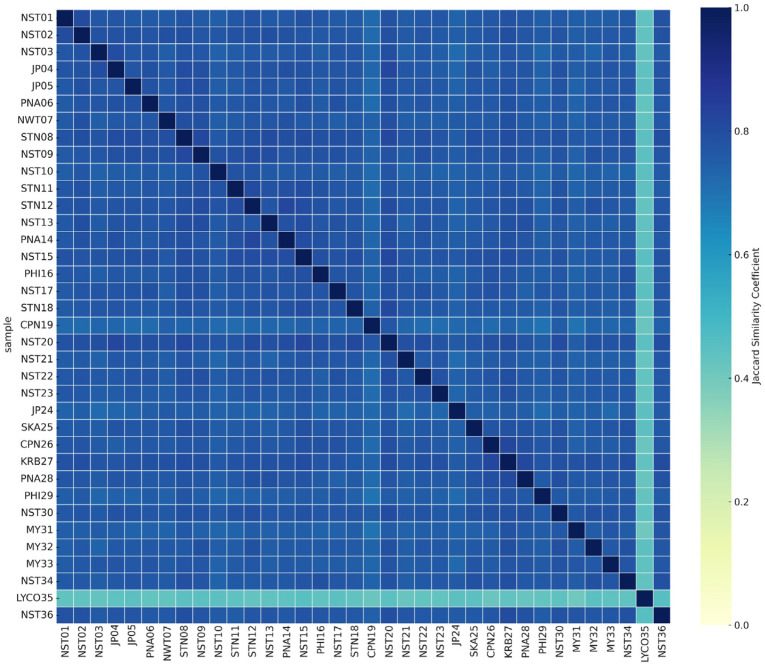
Heatmap of Jaccard similarity coefficients among 36 accessions of *Phlegmariurus* and *Lycopodiella* based on AFLP markers. Color gradients indicate levels of pairwise genetic similarity, ranging from light yellow (low similarity) to dark green–blue (high similarity). The heatmap reveals clustering patterns consistent with the AFLP-based dendrogram and highlights regions of high intra-group similarity as well as marked intergroup divergence.

The heatmap constructed from the Jaccard similarity matrix provided a visual representation of the genetic relationships among the 36 accessions based on AFLP profiles (Figure 4). Color gradients ranged from light yellow (low similarity) to dark green-blue (high similarity), with clustered blocks of samples showing high similarity values. Notably, accessions such as CPN26, KRB27, SKA25, and NST15 formed a tightly grouped block with high pairwise similarity, consistent with their close clustering in the AFLP dendrogram. In contrast, the only *Lycopodiella* accession (LYC035) showed significant genetic divergence, with low similarity to most other samples. The heatmap also revealed intermediate clusters, such as those formed by NST36, PHI29, MY31, and NST30, which exhibited moderate similarity to multiple groups.

#### 2.2.2. Simple Sequence Repeat Analysis (SSR)

Eight out of thirteen SSR primer pairs successfully amplified polymorphic fragments, producing a total of 316 scorable alleles across all 44 SSR loci (Supplementary Figure S2). The number of alleles per locus ranged from 1 to 8, with an average of 5.5 alleles per locus. The PIC values ranged from 0.111 to 0.841, with an average of 0.571. Jaccard’s genetic similarity coefficients among 35 *Phlegmariurus* and *Lycopodiella* accessions ranged from 0.053 to 0.800. The lowest similarity (0.053) was observed between NST17 and LYCO35 (*Lycopodiella*). The highest inter-sample similarity (0.800) was found in several closely related *Phlegmariurus* accessions, such as JP05 and NST23 (Supplementary Table S3).

**Figure 5:**
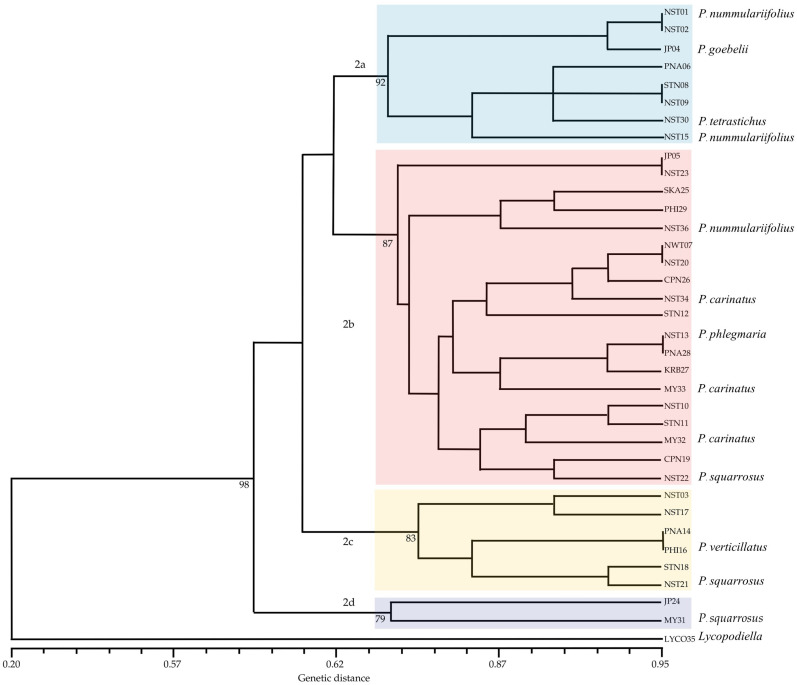
UPGMA dendrogram based on genetic distance calculated from SSR markers among 36 accessions of *Phlegmaiurus* and *Lycopodiella*. The tree was generated using UPGMA clustering and shows four subclusters (2a–2d) with *Phlegmariurus* (Cluster II) and one distinct outgroup (*Lycopodiella*, Cluster I). Bootstrap values (>70) based on 1000 permutations are shown at each node. Note: Accessions not assigned to a specific species were analyzed as part of the *Phlegmariurus* dataset to evaluate genetic variation at the intra-generic level.

The UPGMA dendrogram based on the SSR profile separated the specimens into two major clusters. Cluster I included the outgroup LYCO35 (*Lycopodiella*), while Cluster II contained all 35 *Phlegmariurus* accessions and was subdivided into four subclusters. Subcluster 2a consisted of NST01 (*P. nummulariifolius*), NST02, JP04 (*P. goebelii*), PNA06, STN08, NST09, NST30 (*P. tetrastichus*), and NST15 (*P. nummulariifolius*), which an average Jaccard similarity within this group was 0.386, ranging from 0.118 to 0.800. Subcluster 2b comprised 19 accessions, including JP05, NST23, SKA25, PHI29, NST36 (*P. nummulariifolius*), NWT07, NST20, CPN26, NST34 (*P. carinatus*), STN12, NST13 (*P. phlegmaria*), PNA28, KRB27, MY33 (*P. carinatus*), NST10, STN11, MY32 (*P. carinatus*), CPN19, and NST22 (*P. squarrosus*), for which the average Jaccard similarity was 0.419, ranging from 0.118 to 0.800. Subcluster 2c included NST03, NST17, PNA14, PHI16 (*P. verticillatus*), STN18, and NST21 (*P. squarrosus*), for which the average Jaccard similarity was 0.383, ranging from 0.053 to 0.778. Subcluster 2d consisted of JP24 and MY31 (*P. squarrosus*), sharing a Jaccard similarity value of 0.417 (Figure 5).

**Figure 6:**
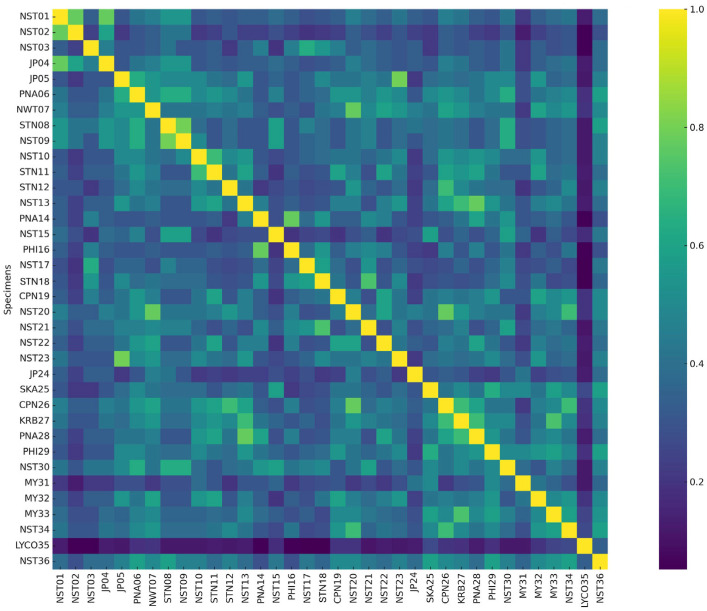
Heatmap of pairwise genetic similarity coefficients among 35 *Phlegmariurus* and one *Lycopodiella* accession based on SSR markers. The Jaccard similarity index was used to assess genetic relationships, with warmer colors (yellow–green) indicating higher similarity and cooler colors (blue–purple) representing greater genetic divergence. The clustering pattern observed here is consistent with the UPGMA dendrogram, reinforcing the presence of distinct intra-generic groupings within *Phlegmariurus*. The outgroup (*Lycopodiella*, LYCO35) shows notably low similarity with all *Phlegmariurus* accessions.

A heatmap, constructed from the Jaccard similarity matrix of all SSR markers, visually presents the genetic relationships among 35 *Phlegmariurus* and one *Lycopodiella* accession. Warmer colors (yellow to green) indicated higher similarity values, while cooler colors (blue to purple) signified lower similarity coefficients. Distinct blocks of high similarity were visible, particularly among accessions in Subcluster 2a (e.g., NST01, NST02, NST15, NST30). In contrast, accessions such as NST17 and JP24 exhibited lower overall similarity with other samples, marked by darker cells in the heatmap. The lowest similarity was recorded between NST17 and LYCO35 (0.053). Meanwhile, accessions with the highest pairwise similarity (0.800) appeared within Subcluster 2a (Figure 6).

#### 2.2.3. Efficiency of AFLP and SSR Markers for *Phlegmariurus* Diversity Assessment

AFLP and SSR markers were compared for *Phlegmariurus* genetic diversity. AFLP (10 primers) yielded 974 loci (95% polymorphic), averaging 92.6 polymorphic bands/assay, with an expected heterozygosity of 0.33, an effective multiplex ratio of 92.6, and a marker index of 28.00. SSR (8 primers) detected 44 (100% polymorphic) bands, averaging 5.5 bands/assay, with a heterozygosity of 0.35, an effective multiplex ratio of 5.5, and a marker index of 1.58 (Table 4).

### 2.3. Molecular Phylogenetics of Phlegmariurus Based on Chloroplast rbcL and psbA-trnH Sequences

#### 2.3.1. Ribulose-1,5-Bisphosphate Carboxylase/Oxygenase Gene (rbcL)

**Figure 7:**
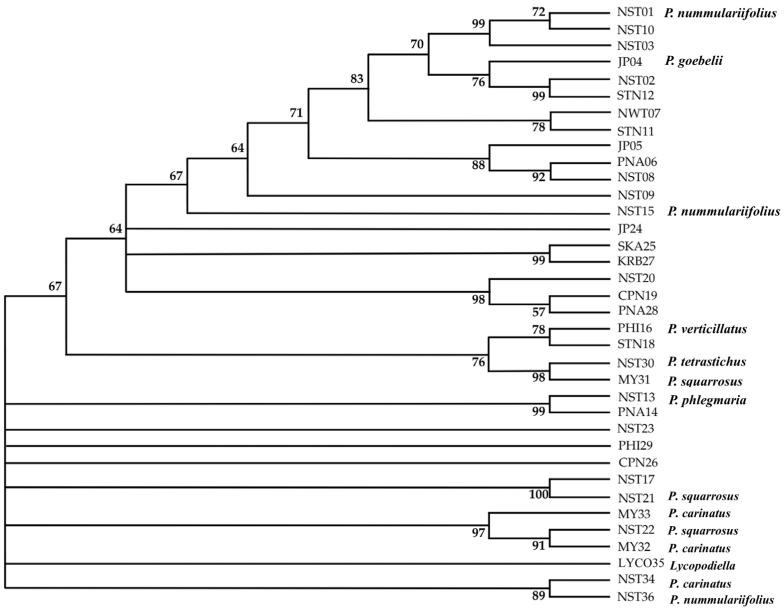
Maximum Likelihood phylogenetic tree of *Phlegmariurus* species based on the *rbcL* coding region. The analysis was performed in MEGA12 using the T92+I substitution model [28], with 1000 bootstrap replicates. Bootstrap values ≥ 50% are shown at the nodes. The final alignment included 36 sequences and 1308 positions after applying the partial deletion option. *Lycopodiella* (sample LYCO35) was used as an outgroup to root the tree. Taxonomic identifications based on morphology are indicated next to each sample.

PCR amplification using *rbcL* primers successfully generated single, distinct bands from all 36 samples. The purified PCR products exhibited sequence similarity ranging from 97.0% to 98.0% (Supplementary Figure S3, Supplementary Table S4, accession number C_AA107151.1-C_AA107186.1). After applying the partial deletion option to remove positions with less than 95% site coverage, the final dataset consisted of 1308 nucleotide positions. The maximum likelihood (ML) tree (log-likelihood = −3170.02) resolved well-supported clades (bootstrap ≥ 70%), aligning with taxonomic expectations. A strongly supported clade (bootstrap = 99%) grouped NST01, NST10, and NST03 as *P. nummulariiifolius*, consistent with morphology, while NST15 clustered nearby with moderate support (67%). JP04 (*P. goebelii*) and JP05 formed a clade with PNA06, NST08, NST09, and NST15 (bootstrap 88%), indicating a moderate association with *P. nummulariifolius* rather than *P. verticillatus*. Additional clades matched species assignments, such as MY32, MY33, and NST34 are strongly supported *P. carinatus* (bootstrap 97%) and NST36, identified as *P. nummulariifolius* (bootstrap 89–97%), NST13 and PNA14 (*P. phlegmaria*, bootstrap 99%), and NST21–NST22 (*P. squarrosus*), which appeared in distinct, well-supported lineages (Figure 7).

#### 2.3.2. *psbA*-*trnH* Intergenic Spacer

**Figure 8:**
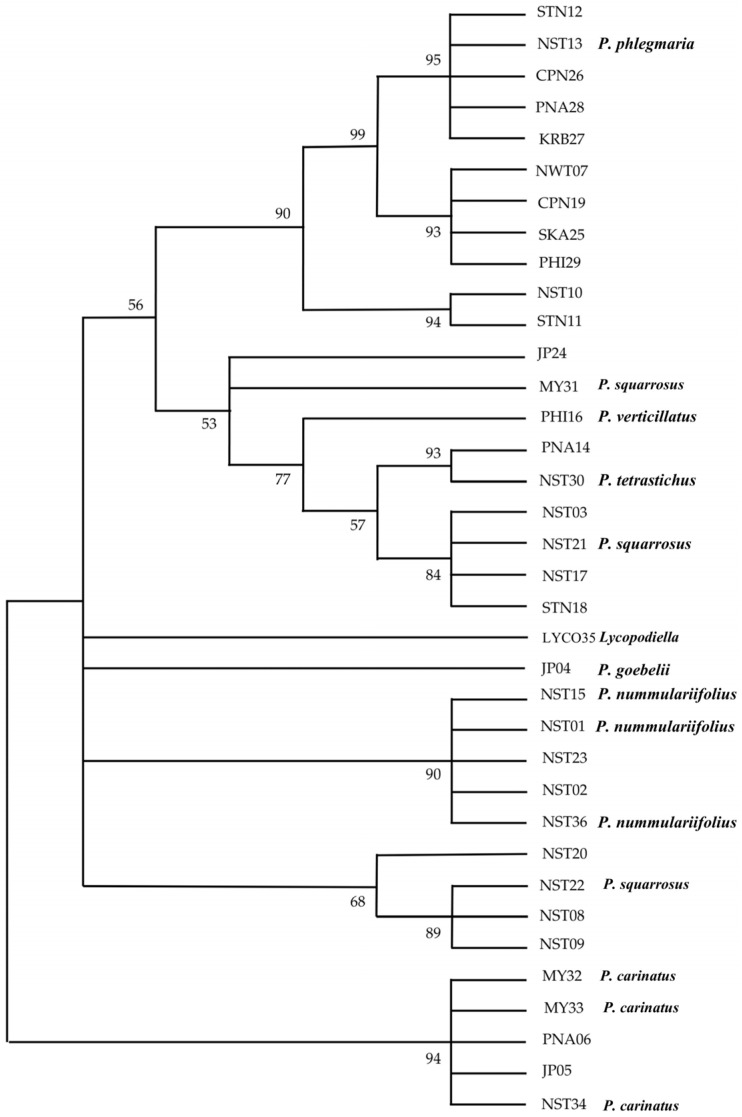
Maximum Likelihood phylogenetic tree of *Phlegmariurus* species and *Lycopodiella* based on the *psbA–trnH* intergenic spacer sequences. The analysis was performed in MEGA12 using T92+I nucleotide substitution model [28]. Bootstrap values ≥ 50% are indicated at the nodes. The dataset comprised 36 nucleotide sequences and a total of 308 aligned positions after applying the partial deletion option. *Lycopodiella* (sample LYCO35) was used as an outgroup to root the tree.

High-quality amplification of *psbA-trnH* was achieved for all specimens, yielding distinct single bands. BLAST comparisons with GenBank references confirmed sequence identities between 87% to 100%, supporting the reliability of the generated data for downstream phylogenetic analysis (Supplementary Figure S4, Supplementary Table S4, accession number C_AA107115.1-C_AA107150.1), comprising 308 nucleotide positions with bootstrap values from 1000 replicates. A well-supported clade (bootstrap = 95–99%) included STN12, NST13, CPN26, PNA28, KRB27, NWT07, CPN19, SKA25, and PHI29, corresponding to *P. phlegmaria*. NST10 and STN11 formed a distinct clade (94% support), likely indicating a cryptic species. MY32, MY33, NST34, PNA06, and JP05 are grouped within *P. carinatus*. Another clade comprised NST15, NST01, NST23, NST02, and NST36 and was identified as *P. nummulariifolius*, supported by morphology. NST22 and NST21, both assigned to *P. squarrosus*, were not grouped together, while JP04 (*P. goebelii*) was distant from its expected relatives. The outgroup *Lycopodiella* was clearly separated, validating tree rooting (Figure 8).

### 2.4. Descriptions of Three New Phlegmariurus Species, NST20, NST02, and STN11

Results from the morphological classification of *Phlegmariurus* species, combined with molecular marker data, identified NST20, NST02, and STN11 have been classified as new species of *Phlegmariurus* sp. Descriptions of NST20, NST02, and STN11 are provided below.

**NST02 *Phlegmariurus* sp. Hang Hong**

**Figure 9:**
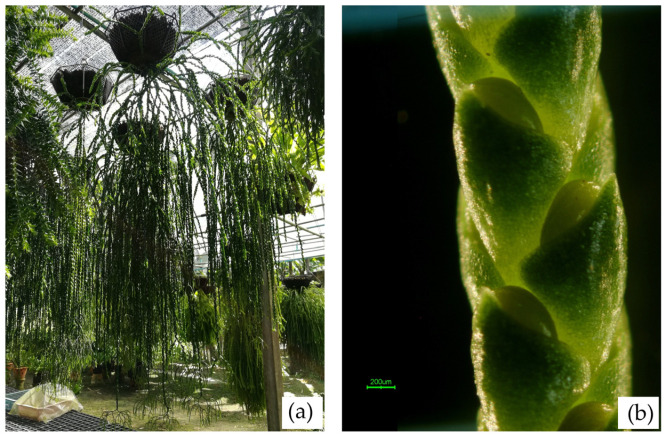
NST02 (Hang Hong). (**a**) An epiphytic plant from Khao Luang National Park, Nakhon Si Thammarat. (**b**) Part of a strobilus showing sporophylls and sporangia.

Type. Epiphytic plant from Nakhon Si Thammarat, Thailand (Figure 9a).

Description. Medium–sized, tufted epiphyte. **Stem:** pendulous or semi–erect, 150 cm or more long, 2–4 mm in diameter without leaves, 0.4–0.6 cm wide across the leaves, green, branched once or sometimes twice towards the apex. **Leaves:** crowded, ovate–lanceolate, apex rounded to acute, base truncated, about 1.1–1.3 cm × 0.4–0.6 cm, margin entire, midrib distinct on both surfaces. **Strobilus:** green or yellowish-green, in pairs, each branching dichotomously once or twice or more forked, (1.0–) 1.6–10 (–11) cm long, distinct and abruptly slender at the apex of vegetative branches (Figure 9b). **Sporophylls:** ovate, apex short acuminate, usually not covering the whole sporangium when mature, appressed, ovate with acute apex, to 1 mm long, 1.5 mm broad, abruptly narrowed above the sporangium and usually only partly covering the mature sporangium.

**STN11 *Phlegmariurus* sp. Chong Baisorn Satun**

**Figure 10:**
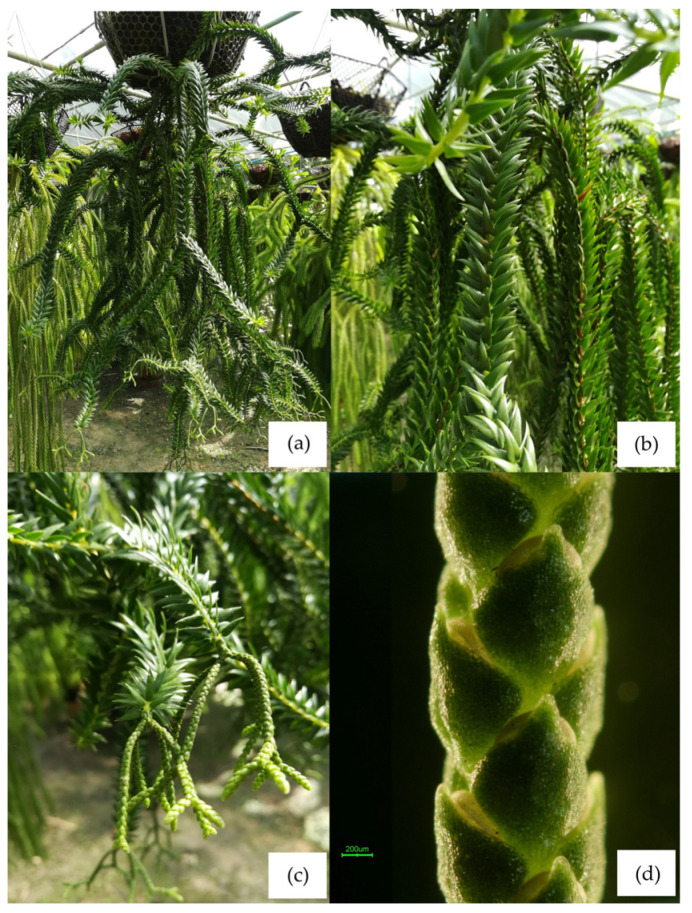
STN11 (Chong Baisorn Satun). (**a**) An epiphytic plant from Satun. (**b**) microphylls narrowly lanceolate, sessile base. (**c**) sporophylls crowded to subdistinct, ovate-subdeltoid. (**d**) part of Strobilus showing sporophylls usually only partly covering the mature sporangium.

Type. Epiphytic plant from Satun, Thailand (Figure 10a).

Description. Epiphytic, repeatedly forked. **Stem** 45–60 cm or more long, 4–12 mm in diameter without leaves, pendulous, one to three times dichotomously forked. **Leaves** narrowly lanceolate (Figure 10b), elliptic to ovate, apex acute to rounded, narrowing towards sessile base, patent, about 0.4–1.3 cm × 0.4–0.6 cm, chartaceous to slightly succulent in texture, margins entire, midrib more or less distinct on lower surface. **Strobili** terminal, cylindrical, distinctly demarcated from vegetative parts of the stem, repeatedly dichotomously branched. **Sporophylls** crowded to sub–distinct, ovate–subdeltoid (Figure 10c), about 1.0 mm × 1.2 mm, entire, green, usually only partly covering the mature sporangium (Figure 10d).

**NST20 *Phlegmariurus* sp. Chong Nakarat**

**Figure 11:**
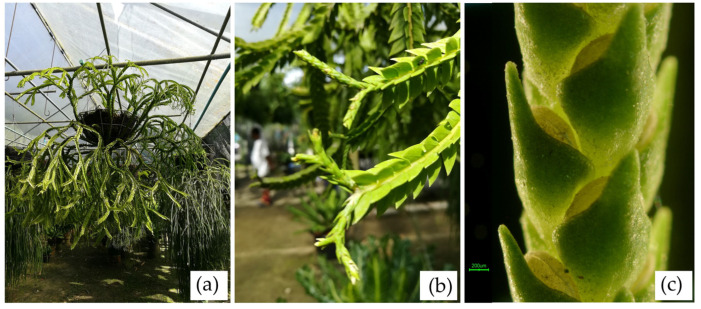
NST20 (Chong Nakarat). (**a**) an epiphytic plant from Nakhon Si Thammarat. (**b**) a branch with strobili. (**c**) part of Strobilus showing sporophyll, sporangium borne at the base of the sporophyll.

Type. Epiphytic plant from Nakhon Si Thammarat, Thailand (Figure 11a).

Description. Epiphytic, repeatedly forked. **Stem** pendulous, 1–4 times branching into two equal branches at irregular intervals, brown, dark, and lustrous in the oldest parts, paler near the growing point, coarse. **Leaves** narrowly lanceolate, elliptic to ovate, apex acute to rounded, narrowing towards sessile base, patent, about 9–15 mm × 9–9 mm, chartaceous to slightly succulent in texture, margins entire, midrib more or less distinct on lower surface. **Strobili** terminal, cylindrical, distinctly demarcated from vegetative parts of the stem, repeatedly dichotomously branched (Figure 11b). **Sporophylls** crowded to subdistant, appressed, ovate-subdeltoid, entire, green, turning yellowish at maturity, only partly covering the sporangium. Sporangium borne at the base of the sporophyll, reniform, deeply grooved, sessile (Figure 11c).

## 3. Discussion

*Phlegmariurus* is an ancient genus of plants, commonly known as firm clubmosses, and is admired for its ornamental value due to a variety of beautiful leaf arrangements. Moreover, they are of significant interest due to their medicinal alkaloids, especially for their potential in treating Alzheimer’s disease [29,30]. The genus *Phlegmariurus* has been frequently reclassified, leading to ongoing uncertainty in its identification. The complex and evolving taxonomy of this family requires further research, as misidentification can impact medicinal use. Current studies on *Phlegmariurus* genetic variation use molecular techniques to clarify species relationships, which is crucial for effective biodiversity conservation and impacts their practical applications.

Multivariate analysis of 15 morphological traits from 27 accessions revealed clear differentiation between *Phlegmariurus* and *Lycopodiella*, with *Phlegmariurus* exhibiting more robust vegetative structures, upright growth forms, and distinctive qualitative traits such as leaf angle and apex shape. In contrast, *Lycopodiella* displayed creeping habits and narrower morphometric profiles, supporting their taxonomic separation [1,2,3,10].

Quantitative traits effectively grouped accessions by variety and aligned with taxonomic classifications. Analysis of 409 herbarium specimens across seven species revealed three major groups within Lycopodiaceae, distinguished by sporangium shape, leaf thickness, and shoot diameter [2]. PCA emphasized diagnostic features such as leaf orientation and apex shape, consistent with earlier studies on Southeast Asian lycophytes [2,5].

Within *Phlegmariurus*, PCA and cluster analyses identified distinct subclusters, though many accessions overlapped along PC1, indicating limited differentiation among key morphological traits. However, outliers like NST02, STN11, and NST20 consistently occupied peripheral positions in ordination space and dendrograms. NST02 was characterized by robust stems and long fertile branches, while STN11 and NST20 displayed unique apex shapes and microphyll dimensions. These morphotypes align with cryptic diversity documented in Neotropical *Phlegmariurus* [10,31].

Notably, STN11 exhibited exceptional fertile branch length and a distinct leaf apex—traits previously shown to be taxonomically informative in *Phlegmariurus* [32]. This supports Øllgaard’s [8] identification of cryptic lineages and aligns with synapomorphic apex traits found in global plastid phylogenies [31]. Similar morphological criteria have been used to define new Asian species, including *P. yunfengii* and *P. shingianus* in China [33], and *P. iminii* in Peninsular Malaysia [34]. Nevertheless, convergent evolution in apex and branching morphology—well documented in Neotropical taxa—highlights the limitations of relying solely on morphology for species delimitation [31].

Geographical origin also influenced clustering, particularly in *Phlegmariurus*, reflecting broader biogeographic patterns within *Lycopodiaceae* [2,9]. However, environmental plasticity complicates classification based purely on morphological traits [8,10]. While branching patterns and leaf morphology effectively separated the genera *Phlegmariurus*, *Lycopodiella*, and *Lycopodium* [3], morphological homoplasy often reduced resolution at the species level.

Although microphyll size (length and width) is generally considered taxonomically informative in lycophyte classification [2], the PCA results in this study showed relatively low PC1 loadings for microphyll length and width. This suggests that these traits contributed minimally to the primary axis of morphological variation among the studied *Phlegmariurus* specimens. However, other leaf-related traits, such as the distance from apex to the widest portion of the microphyll, showed a much higher loading and may serve as more informative characters for distinguishing taxa.

Overall, while morphology provided valuable insight into genus-level distinctions and identified potentially novel lineages, it was insufficient for fine-scale delimitation. Integrative taxonomic approaches that incorporate molecular, anatomical, and phytochemical data are essential to resolve the complexity of species boundaries within *Lycopodiaceae* [3,10].

AFLP-based UPGMA analysis of *Phlegmariurus* and *Lycopodiella* demonstrated a high level of polymorphism (95.07%) and a mean PIC value of 0.32, with 34.5% of loci exceeding 0.30, indicating the high discriminatory capacity of AFLP in evaluating lycophyte genetic diversity. This aligns with *Spenceria ramalana* [35], where 58.85% of variation was attributed to polymorphic fragments. Compared to *Osmanthus fragrans* [19], where eight AFLP primer pairs detected 86.8% polymorphic bands and similarity coefficients of 0.69 to 0.87. The high genetic diversity in *Phlegmariurus* and *Lycopodiella* likely stems from evolution, outcrossing, or ecological adaptation. *P. carinata* suggests gene flow or shared ancestry, while geographic isolation drives variation. Like *O. fragrans* [19], genetic relationships were shaped by geography and evolution. Both studies confirm AFLP’s effectiveness in assessing population structure and genetic divergence. Despite this difference, the AFLP method remains effective for distinguishing genetic differences. Our cluster analysis grouped samples into seven clusters, similar to how AFLP divided chili genotypes into distinct clusters. The GS values in our study, ranging from 0.410 to 0.818, align with GS values found in chili studies (0.19 to 0.85 in Taiwan and 0.24 to 0.90 in Indian genotypes) [36], supporting the utility of AFLP in genetic research.

Heatmap visualization based on AFLP-derived Jaccard coefficients effectively illustrated genetic clustering among the 36 accessions, supporting dendrogram results and revealing distinct genetic divergences. These findings are consistent with previous AFLP-based diversity studies. These results are consistent with prior studies utilizing AFLP and heatmap analyses to explore genetic diversity. Comparable approaches have been employed in other species, yielding similar insights. In *Carpinus tientaiensis*, AFLP analysis showed that most genetic variation occurred within populations, with inter-population differences associated with geographic and environmental factors [37]. In *Sporothrix* spp., AFLP revealed high intraspecific variation, enabling clear separation of strains into distinct genetic groups [38].

Our study of *Phlegmariurus* and *Lycopodiella* using 44 polymorphic SSR markers revealed substantial genetic variation. This is supported by the overall GS values ranging from 0.68–0.95, with a narrower range within *Phlegmariurus* (0.78–0.94). The analysis of these 44 SSR loci identified 316 alleles, averaging 5.5 alleles per locus, and yielded a high mean PIC value of 0.571 (range 0.11–0.84), confirming the effectiveness of these SSR markers for genetic diversity assessment in lycophytes. The power of these markers in resolving genetic structure was further demonstrated by the Jaccard similarity and UPGMA analysis, which effectively separated the 36 accessions into two main clusters (GS = 0.68), highlighting their capacity to delineate relationships within and between species. When compared to other SSR marker studies, our findings show higher allelic richness than reported in soybean [39] (mean = 2.79 alleles per locus, average PIC of 0.44), while a study on non-heading Chinese cabbage [40] with more markers showed higher genetic diversity (PIC = 0.555–0.911). The effectiveness of SSRs in related lycophytes is also supported by findings in endangered *H. serrata* [22], which revealed high allelic variation.

The power of SSR marker analysis in elucidating genetic relationships was further enhanced by heatmap visualization, providing a detailed view of the genetic structure among the 36 accessions. The heatmap corroborated the groupings observed in the UPGMA dendrogram, while also revealing finer-scale genetic distinctions. For instance, the high similarity within Subcluster 2a and the distinct profiles of NST17 and JP24 were clearly visualized. Notably, the lowest similarity (0.053) observed between NST17 (*Phlegmariurus*) and LYC035 (*Lycopodiella*) on the heatmap underscores the significant divergence between these two genera, a finding consistent with the broader clustering analysis. The utility of integrating SSR markers with heatmap visualization for assessing genetic diversity and relationships is well-supported by previous research. Studies on *Camellia nitidissima* var. *phaeopubisperma* [39], *Juglans regia* [41], and *Hemerocallis* spp. [42] have similarly demonstrated the effectiveness of this combined approach in revealing genetic patterns, clustering genotypes, and identifying breeding lines. These findings, consistent with our results, highlight the value of this integrated methodology for enhancing the resolution of genetic diversity assessments and informing conservation and breeding strategies in various plant species.

While our study leveraged 44 polymorphic SSR markers to elucidate genetic relationships within *Phlegmariurus* and *Lycopodiella*, different marker systems offer complementary strengths. AFLP studies in these genera demonstrate broader genomic coverage with more polymorphic bands (926) and a higher marker index (28.00), ideal for detecting large-scale differentiation. Our SSR analysis, though yielding fewer loci with lower marker index (1.58), provided higher expected heterozygosity (0.35 vs. 0.33) and co-dominant inheritance, enabling finer resolution of closely related accessions as shown in our UPGMA dendrogram and heatmap. Similar complementary patterns between AFLP and SSR markers have been observed in *Phaseolus vulgaris* [43,44]. While AFLP offers efficient genome-wide screening with dominant inheritance limitations, our SSR data’s higher heterozygosity suggests potential ongoing gene flow within *Phlegmariurus*, providing unique insights into lycophyte genetic dynamics.

The comparative analysis of phylogenetic trees based on the *rbcL* coding region and the *psbA–trnH* intergenic spacer highlights their distinct abilities to resolve species-level relationships within *Phlegmariurus*. While both markers are widely used in plant DNA barcoding and phylogenetics, their effectiveness varies due to differences in evolutionary characteristics. The *rbcL* gene, being highly conserved, resolved higher-level relationships effectively, grouping major clades such as *P. nummulariifolia*, *P. carinata*, and *P. phlegmaria* with strong bootstrap support (89–99%). However, its low nucleotide variability limits its ability to distinguish closely related species, as suggested by previous studies [45,46], indicating that *rbcL* alone may not be sufficient for species delimitation in rapidly diverging lineages. In contrast, the *psbA–trnH* spacer exhibited greater polymorphism, including indels and mutations, making it more effective for resolving species-level relationships. This marker successfully distinguished *P. squarrosa* accessions and clustered samples based on morphological traits [47,48]. Its fast-evolving nature and higher sequence variability allowed for the clear separation of species such as *P. squarrosa*, *P. carinata*, and *P. tetrasticha*, and also captured both inter- and intra-specific diversity. This shows that *psbA–trnH* is particularly valuable for species identification and fine-scale population studies in taxonomically complex groups. While *rbcL* is suitable for establishing the phylogenetic backbone, *psbA–trnH* enhances resolution at terminal branches, making it essential for integrative phylogenetic and taxonomic studies of *Phlegmariurus* and related taxa [49].

Despite their utility, alignment ambiguities in non-coding spacers and potential plastid capture events necessitate cautious interpretation. Incorporating additional plastid (e.g., matK, rps4) and nuclear markers (e.g., ITS) can improve species delimitation by reducing single-locus bias and enhancing phylogenetic resolution [50]. The combination of *rbcL* and *psbA–trnH* offers complementary strengths—*rbcL* resolves deeper relationships, while *psbA–trnH* captures finer-scale divergence.

Overall, the combination of *rbcL* and *psbA–trnH* provides a comprehensive approach, with *rbcL* establishing high-level relationships and *psbA–trnH* offering valuable resolution at finer scales, essential for detailed species-level analysis. Similarly, phylogenetic analyses of 1150 DNA sequences from seven plastid markers (*atpA*, *psbA*-*trnH*, *rbcL*, *rps4*, *rps4*-*trnS*, *trnL*, *trnL*-*F*), identifying three subfamilies (*Huperzioideae*, *Lycopodioideae*, and *Lycopodielloideae*) and 17 major clades. *Lycopodiella serpentina* is sister to *Palhinhaea*, leading to the new genus *Brownseya*. Key genera (*Huperzia*, *Lycopodiella*, *Pseudolycopodiella*, and *Spinulum*) are monophyletic, and spore morphology supports phylogenetic relationships [51].

In this study, an integrative approach combining morphological clustering with AFLP, SSR, *rbcL*, and *psbA–trnH* markers was applied to clarify phylogenetic relationships within *Phlegmariurus*. Four major clades, *P. nummulariifolia*, *P. carinata*, *P. phlegmaria*, and *P. squarrosa* were consistently recovered across all markers, confirming their genetic distinctness [40,52,53,54,55]. Three previously unclassified accessions (NST02, STN11, NST20) formed well-supported novel lineages, validated by both genome-wide (AFLP) and locus-specific (*psbA-trnH*) data. The combined strengths of broad genomic coverage (AFLP), fine-scale allelic resolution (SSR), deep-branch support (*rbcL*), and terminal-branch discrimination (*psbA-trnH*) provide a robust framework for accurate species delimitation, conservation unit delineation, and marker-assisted cultivar development. Future incorporation of genome-wide SNP datasets (e.g., RAD-seq) and nuclear loci (e.g., ITS) will enhance phylogeographic resolution, clarify reticulation events, and facilitate the discovery of adaptive genetic variation [45].

## 4. Materials and Methods

### 4.1. Plant Materials

The Phlegmariurus specimens used in this study were collected from natural environments across various locations in Southern Thailand, including Khao Luang National Park, Krung Ching National Park (Nakhon Si Thammarat), and additional sites in Phang-nga, Narathiwat, Satun, Chumphon, Songkhla, and Krabi. All specimens, including additional samples from Japan, the Philippines, Malaysia, and the Solomon Islands, were housed in the Phosadet Garden Tropical Nursery for care and maintenance during the study period. These included 36 specimens: 35 *Phlegmariurus* specimens and one outgroup specimen of *Lycopodiella*. Among the *Phlegmariurus* specimens, 13 individuals were identified as belonging to seven known species, including *P. nummulariifolius* (NST01, NST15, NST36), *P. goebelii* (JP04), *P. phlegmaria* (NST13), *P. verticillatus* (PHI16), *P. squarrosus* (NST21, NST22, MY31), *P. tetrastichus* (NST30), and *P. carinatus* (MY32, MY33, NST34) (Supplementary Table S4). Samples were preserved on ice during collection, transferred to the laboratory, and stored in a −20 °C freezer.

### 4.2. Morphology Study of Phlegmariurus

The morphology of *Phlegmariurus* was analyzed both qualitative and quantitative characteristics. For qualitative analysis, five qualitative characters were assessed: leaf shape (1 = linear, 3 = ovate, 5 = lanceolate, 7 = ovate-lanceolate, 9 = elliptic), leaf angle to the stem (1 = 15°, 3 = 45°, 5 = 90°), leaf apex (1 = acute, 5 = acuminate), leaf base (1 = acute, 3 = attenuate, 5 = obtuse), and leaf attachment (1 = sessile, 5 = sub-petiolate).

For quantitative analysis, morphological measurements were taken from fertile branches of each sample. Ten morphological traits were measured, comprising five vegetative traits (main stem diameter, lateral branch diameter, microphyll width, microphyll length, and apex-to-widest point distance of microphyll) and five reproductive traits (fertile branch length, strobilus length and width, and sporophyll length and width). Measurements of stem, branches, microphyll, and strobilus were recorded in centimeters (cm), while measurements of sporophylls and apex-to-widest microphyll distance were recorded in millimeters (mm). All morphological measurements were performed using a standard caliper. The raw dataset was initially screened for missing values. In order to meet the assumptions of multivariate analysis, any specimens with incomplete data were excluded using listwise deletion. As a result, 27 specimens with complete morphometric data were retained for subsequent analyses.

PCA was conducted using JASP version 0.19.3.0 [56]. The PCA was performed using a correlation matrix, with the number of components determined by the Eigenvalue > 1 criterion. In addition, a biplot was constructed to depict the distribution of specimens in PC space and to illustrate the influence of individual traits on group separation.

Hierarchical cluster analysis was performed using Ward’s method and Euclidean distance to explore group structure among specimens based on morphometric similarity. The analysis was conducted in JASP version 0.19.3.0 via the Clustering and Hierarchical Cluster Analysis module. The resulting dendrogram visualized the relative dissimilarity among specimens, with branch lengths reflecting morphometric distances.

### 4.3. Analysis of Genetic Variability Among Phlegmariurus Species Using AFLP and SSR Marker

#### 4.3.1. Total Genomic DNA Extraction

Total genomic DNA was extracted from microphyll using a modified [57] method. About 0.05–0.1 g of tissue was ground in 0.5 mL of extraction buffer (100 mM Tris-Cl, pH 8.0; 20 mM EDTA, pH 8.0; 1.4 M NaCl; 1% PVP, 2% CTAB, and 2% β-mercaptoethanol), followed by adding another 0.5 mL of buffer and incubating at 65 °C for 1–2 h. After centrifugation, the supernatant was purified twice with chloroform, treated with RNaseA, and further purified with chloroform:isoamyl alcohol (24:1). Genomic DNA was quantified using a NanoDrop ND-1000 Spectrophotometer (NanoDrop products, Wilmington, DE, USA) and was quality checked using electrophoresis on a 1% (*w*/*v*) agarose gel. The DNA was stored at −80 °C before further use.

#### 4.3.2. Genetic Variability Among *Phlegmariurus* Species Using AFLP Analysis

AFLP analysis followed a modified method by [20]. 500 ng of genomic DNA was digested with *Eco*RI and *Mse*I, then ligated with ER and MS adaptors. The reaction was incubated at 37 °C for 1 h for digestion and 3 h for ligation. The ligated DNA was diluted 10-fold with 0.1xTE buffer for pre-selective amplification. Pre-selective amplification used ER-N and MS-N primers with an additional 3′ nucleotide (Supplementary Table S5). The reaction included the DNA template, primers, GoTag^®^ flexi buffer, MgCl_2_, dNTPs, and DNA polymerase. The thermal cycle was 20 cycles at 94 °C, 56 °C, and 72 °C. The PCR product was then diluted 10-fold with 0.1xTE buffer for selective amplification. Pre-selective amplification products were further amplified using primers with three selective nucleotides (ER-NNN and MS-NNN). The pre-selective PCR products were diluted 10-fold in 0.1xTE buffer and used as templates. The amplification mixture included 2 µL of template DNA, GoTag^®^ flexi buffer, MgCl_2_, dNTPs, and DNA polymerase, with a final volume of 20 µL. The PCR process involved an initial denaturation at 94 °C for 2 min, followed by 10 cycles with a decreasing annealing temperature and then 30 cycles at a stable temperature. Denaturing loading buffer was added to terminate the reaction. A 5% polyacrylamide gel (19:1 of acrylamide:bis-acrylamide), with 7.5 M urea and 1X TBE buffer, was prepared and cast using the Sequi-Gen GT Cell (Bio-Rad Laboratories, Hercules, CA, USA). After pre-running to 50 °C, the selective amplification product was denatured at 94 °C for 3 min, chilled on ice, and then loaded (5–8 µL) onto the gel. The gel was run at 50 watts and 50 °C for 2 h. The gel was then stained with silver nitrate. The process involved fixing in 10% acetic acid, washing, staining with silver nitrate and formaldehyde, rinsing, and developing with a sodium carbonate solution until bands appeared. The gel was then stopped with acetic acid, washed, and air-dried.

#### 4.3.3. Genetic Variability Among *Phlegmariurus* Species Using SSR Analysis

A search of *Phlegmariurus* sequences on NCBI yielded 1324 nucleotide sequences as of 11 May 2022. These sequences were assembled using Egassembler [58] to create consensus sequences, with redundancy removed. SSRs were identified using WEBSAT (http://wsmartins.net/websat/; accessed on 11 May 2022), and SSR primers were designed with the Oligonucleotide Properties Calculator (https://www.biosyn.com/gizmo/tools/oligo/oligonucleotide%20properties%20calculator.htm; accessed on 11 May 2022) (Supplementary Table S6). PCR reactions were performed in 10 µL volumes using a BioRad Thermal Cycler (Biorad, Sydney, Australia), with an initial denaturation at 95 °C for 15 min, followed by 35 cycles of 95 °C for 30 s, annealing at 50–65 °C for 30 s, and extension at 72 °C for 1 min. A final extension at 72 °C for 10 min was included. PCR products were resolved on a 1.5% agarose gel with 7.5 M urea and 1X TBE buffer at 50 °C and 50 watts for 2 h. The gel was then silver-stained, fixed in 10% acetic acid, washed, and developed with sodium carbonate solution until bands were visible, followed by a final wash and air-drying.

#### 4.3.4. Evaluating the Effectiveness of AFLP and SSR Markers for Genetic Diversity Analysis in *Phlegmariurus*

Data scoring and analysis involved creating profiles for each accession using AFLP and SSR markers, all bands were scored as binary characters (1 = present, 0 = absent). The effectiveness of the two marker techniques was compared by estimating the following metrics: polymorphic bands, monomorphic bands, average polymorphic bands per assay unit, number of loci, loci per assay unit, expected heterozygosity, polymorphic loci fraction, effective multiplex ratio, and marker index [44]. Genetic similarity estimates were calculated using Jaccard’s coefficient [59], and samples were clustered using UPGMA in *NTSYSpc* version 2.0 [60]. Dendrogram accuracy was evaluated via bootstrap with 1000 replications.

### 4.4. DNA Sequencing and Phylogenetic Analysis Using Chloroplast rbcL and psbA-trnH Sequences

Molecular markers were selected based on previous phylogenetic studies of *Lycopodiaceae* [9,21,26,27,61]. Two chloroplast loci were chosen: *rbcL* and *trnH*-*psbA* using primers from published sources. The *rbcL* region was sequenced with primers *rbcL*a-F and *rbcL*a-R, and the *trnH*-*psbA* region with *psbA*–F and *trnH*-R [26] (Supplementary Table S5). PCR was conducted in 10 µL volumes using a BioRad Thermal Cycler. For *rbcL* amplification: 95 °C for 7 min, followed by 30 cycles at 95 °C for 45 s, 55 °C for 70 s, and 72 °C for 50 s, with a final 5 min extension at 72 °C. For *psbA*-*trnH*: 94 °C for 5 min, 25 cycles at 94 °C for 45 s, 55 °C for 45 s, and 72 °C for 50 s, with a final 5 min extension at 72 °C.

PCR products were then purified using the GenePhlow™ Gel/PCR Kit before sequencing at FirstBase Laboratory, Malaysia. Phylogenetic analyses were performed using *MEGA 12* [51] via maximum likelihood methods. The Tamura 3-parameter model with a proportion of invariant sites (T92+I) was selected as the best-fit model based on BIC values using MEGA version 12. This model was applied in subsequent phylogenetic analyses [28]. Internal support was assessed using bootstrap analysis with 1000 replicates [62] and decay indices [63].

## 5. Conclusions

In this study, a comprehensive phylogenetic analysis of *Phlegmariurus* species from Khao Luang National Park, Nakhon Si Thammarat, Thailand, was conducted using a combination of morphological characteristics and molecular markers (AFLP, SSR, *rbcL*, and *psbA*-*trnH*). Morphometric analyses (PCA and hierarchical clustering) revealed distinct morphotypes, confirming the presence of seven previously described species. Molecular results further supported these groupings and successfully differentiated *Phlegmariurus* from *Lycopodiella*. The phylogenetic trees constructed from the four molecular markers showed consistent clustering patterns, reinforcing the reliability of these markers in species identification. For example, AFLP analysis revealed distinct groupings of *P. nummulariifolius*, *P. goebelii*, and *P. phlegmaria*, which were also supported by the *rbcL* and SSR trees. The *psbA*-*trnH* marker further distinguished species like *P. nummulariifolius*, *P. tetrastichus*, and *P. squarrosus*, with high genetic similarity observed within certain subclades, indicating close evolutionary relationships. This alignment of findings across different molecular markers validates the integrated approach used in this study and enhances the robustness of our species identification efforts. Moreover, this study highlights the potential for discovering new cultivars within the *Phlegmariurus* genus due to its high intraspecific variability, likely driven by its ability to propagate through highly germinable spores. The identification of distinct genetic lineages could inform the cultivation of novel varieties, with significant implications for the ornamental plant market. These findings underscore the importance of continued research into the genetic diversity of *Phlegmariurus*, which could lead to the discovery of new, commercially valuable cultivars and contribute to the broader understanding of plant systematics and conservation.

## Figures and Tables

**Table 1:** Means and standard deviation of ten quantitative characters of the *Phlegmariurus* and *Lycopodiella* genera.

Characters	*Phlegmariurus*		*Lycopodiella*	
	Mean (cm)	±SD	Mean (cm)	±SD
Diameter of main stem	4.17	2.40	0.60	0.00
Diameter of lateral branch	0.65	0.71	0.43	0.00
Width of microphyll	0.40	0.22	0.30	0.00
Length of microphyll	1.00	0.55	0.42	0.00
Distance from apex to the widest portion of microphyll (mm)	0.44	0.22	0.30	0.00
Length of fertile branches	12.60	14.92	1.20	0.00
Length of strobilus	8.41	4.11	0.21	0.00
Width of strobilus	0.33	0.27	0.08	0.00
Length of sporophyll (mm)	0.43	0.31	1.37	0.00
Width of sporophyll (mm)	0.29	0.31	0.13	0.00

**Table 2:** PCA Loading of ten quantitative and five qualitative morphological traits in *Phlegmariurus*.

Trait	PC1 Loading	PC2 Loading
Distance from apex to the widest portion of microphyll (mm)	−0.99231	0.034427
Width of microphyll (cm)	−0.08033	−0.02468
Leaf attachment (ordinal scale)	−0.0522	−0.22074
Leaf angle of stem (ordinal scale)	−0.04771	−0.06406
Diameter of lateral branch (cm)	−0.03444	0.196136
Leaf base (ordinal scale)	−0.02633	−0.13795
Leaf apex (ordinal scale)	−0.02567	−0.56209
Width of strobilus (cm)	−0.02043	0.075919
Length of microphyll (cm)	−0.01587	0.043359
Length of fertile branches (cm)	−0.01199	−0.54215
Length of strobilus (cm)	−0.00559	0.121033
Width of sporophyll (mm)	−0.00464	−0.28928
Diameter of main stem (cm)	−0.00462	0.223104
Leaf shape (ordinal scale)	0.001072	−0.32471

Note: ordinal traits were scaled based on qualitative coding as defined in the methods.

**Table 3:** AFLP analysis of primer combination among 35 *Phlegmariurus* sp. and *Lycopodiella*.

Primer Combination		Number of Total Loci Detected	Number of Polymorphic Loci	% of Polymorphic Loci
E-AA	M-CAG	92	92	100
E-AG	M-CGA	133	129	96.9
E-AG	M-CGT	85	73	85.9
E-AT	M-CCA	88	83	94.3
E-AT	M-CTA	96	91	94.8
E-AT	M-CGA	82	69	84.1
E-ACC	M-CGT	107	104	97.2
E-AGA	M-CCA	108	105	97.2
E-AGA	M-CGT	71	71	100
E-AGC	M-CGT	112	109	97.3
Total		974	926	-

**Table 4:** Comparison of polymorphism level and discriminating capacity of AFLP and SSR markers across 35 *Phlegmariurus* and one *Lycopodiella* accessions.

Index	Marker System	
	AFLP	SSR
Number of assay units	10.00	8.00
Number of polymorphic bands	926.00	44.00
Number of monomorphic bands	48.00	0
Average number of polymorphic bands per assay unit	92.60	5.50
Number of loci	974.00	44.00
Number of loci per assay unit	97.40	5.50
Expected heterozygosity of the polymorphic loci	0.33	0.35
Fraction of polymorphic loci	0.95	1.00
Effective multiplex ratio	92.60	5.50
Marker index	28.00	1.58
Average PIC Value	0.32	0.57

## Data Availability

The dataset generated and analyzed during the current study are available in the National Genomics Data Center’s GenBase repository (http://ngdc.cncb.ac.cn/genbase/; accessed on 18 April 2025) and are publicly accessible under the following accession no. for Thai *Phlegmariurus rbcL* sequences (C_AA107151.1-C_AA107186.1), and Thai *Phlegmariurus psbA*-*trnH* intergenic spacer (C_AA107115.1-C_AA107150.1).

## Supplementary Materials

The following supporting information can be downloaded at: https://www.mdpi.com/article/10.3390/plants14091400/s1, Figure S1. AFLP profile illustrating genetic variations among 35 *Phlegmariurus* sp. and one *Lycopodiella* outgroup. The analysis used *Eco*RI and *Mse*I restriction enzymes with the following primer combinations: (a) E-AA/M-CAG, (b) E-AG/M-CGA, (c) E-AG/M-CGT, (d) E-AT/M-CCA, (e) E-AT/M-CTA, (f) E-AT/M-CGA, (g) E-ACC/M-CGT, (h) E-AGA/M-CCA, (i) E-AGA/M-CGT, (j) E-AGC/M-CGT. Fragments were separated on a 5% polyacrylamide gel and visualized by silver staining (Figure S2). SSR profile illustrating the genetic diversity and relationships among 35 *Phlegmariurus* sp. and one *Lycopodiella* as outgroup. Accessions were amplified using the following primer: (a) Hu-C4, (b) Hu-M6, (c) Hu-M7, (d) Hu-M10, (e) Hu-M11, and (f) Hu-M12. The banding patterns represent the allelic composition at the SSR locus and Figure S3. BLAST alignment results showing 97.0–99.0% identity of *rbcL* sequences from 35 *Phlegmariurus* and *Lycopodiella* accessions matching *P. phlegmaria*, *P. carinatus*, *P. squarrosus*, and *Huperzia phyllantha* compared to published reference sequences in the NCBI GenBank database, Figure S4. BLAST similarity analysis of *psbA*-*trnH* sequences from 35 *Phlegmariurus* and one *Lycopodiella* compared to reference sequences from the NCBI database. The sequences matching *P. carinatus*, *P. squarrosus*, *Huperzia phyllantha*, and *P. pinifolius*, with percentage identity (% Identity) ranging from 94.97% to 100%. Query cover ranged from 87% to 99%, and E-values were all highly significant (e-value < 1e−152), Table S1. Qualitative characters of fertile plants of *Phlegmariurus* sp. and *Lycopodiella* with their methods of scoring, Table S2. Jaccard’s genetic similarity coefficient matrix for 35 *Phlegmariurus* and one *Lycopodiella* accession based on AFLP markers, Table S3. Jaccard’s genetic similarity coefficient matrix for 35 *Phlegmariurus* and one *Lycopodiella* accession based on SSR markers, Table S4. GenBase accession number *Phlegmariurus* specimens and one *Lycopodiell* as outgroup used in this study, Table S5. Primers and adapter sequences used for AFLP analysis, Table S6. SSR primers were used for polymorphism analysis.
