# Supplementary material for: Identification of Indigenous Thai Phlegmariurus Genotypic Population by Integrating Morphological and Molecular Studies
Source: Plants (Basel). 2025 May 7;14(9):1400. doi: 10.3390/plants14091400 (PMC12073110; doi:10.3390/plants14091400)
Supplement: Supplementary file 1 [file plants-14-01400-s001.zip › plants-3542031-supplementary.pdf]

2.2.1).

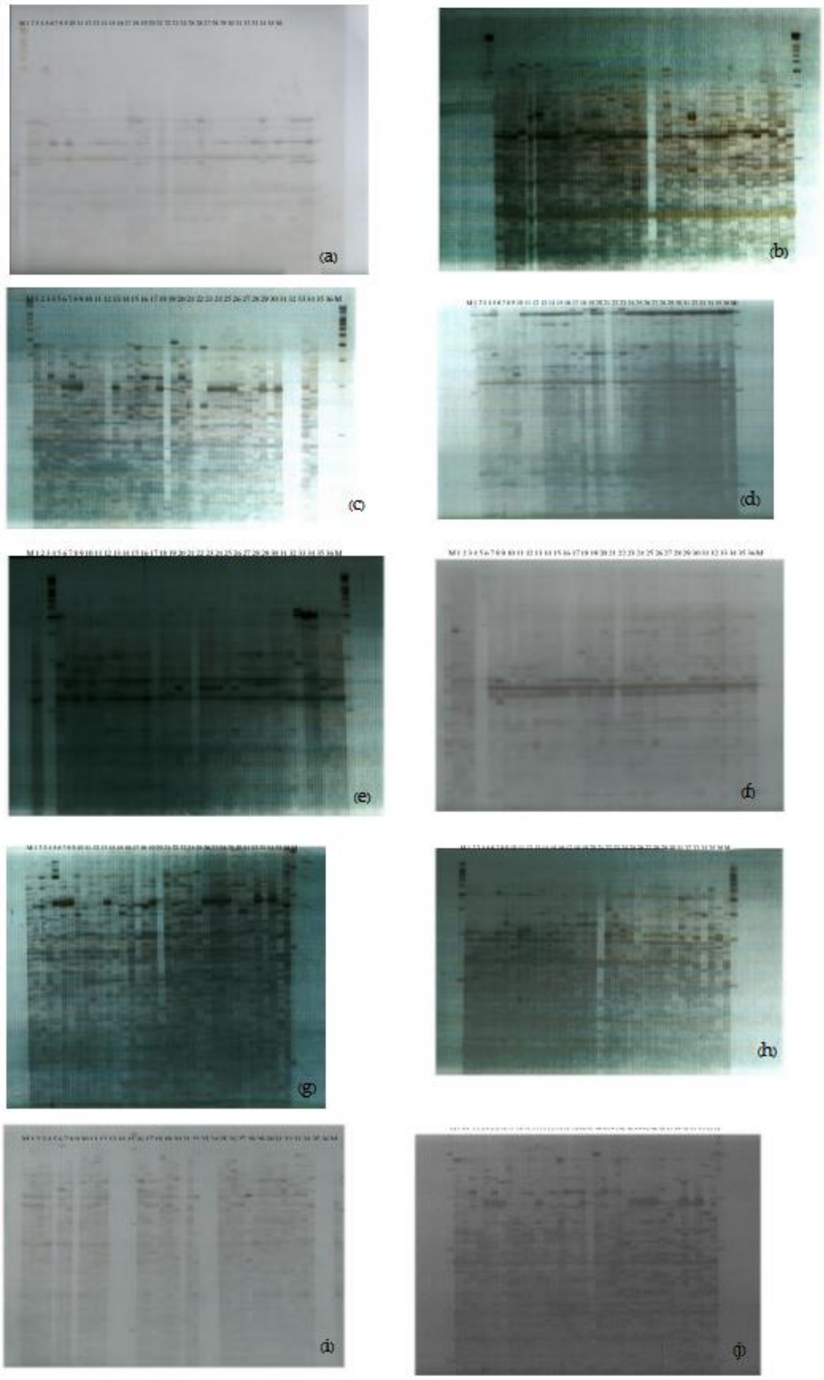

Supplementary Figure S1. AFLP profile illustrating genetic variations among 35 *Phlegmariurus* sp. and one *Lycopodiella* outgroup. The analysis used *Eco*RI and *Mse*I restriction enzymes with the following primer combinations: (a) E-AA/M-CAG, (b) E-AG/M-CGA, (c) E-AG/M-CGT, (d) E-AT/M-CCA, (e) E-AT/M-CTA, (f) E-AT/M-CGA, (g) E-ACC/M-CGT, (h) E-AGA/M-CCA, (i) E-AGA/M-CGT, (j) E-AGC/M-CGT. Fragments were separated on a 5% polyacrylamide gel and visualized by silver staining (Section 2.2.1).

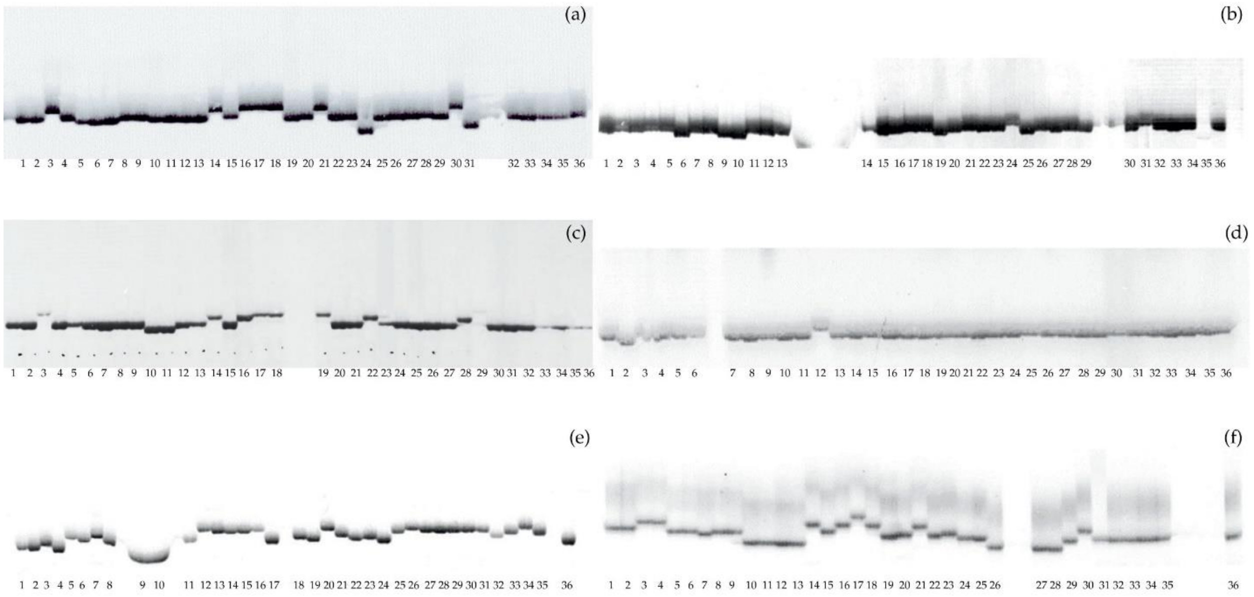

Supplementary Figure S2. SSR profile illustrating the genetic diversity and relationships among 35 *Phlegmariurus* sp. and one *Lycopodiella* as outgroup. Accessions were amplified using the following primer: (a) Hu-C4, (b) Hu-M6, (c) Hu-M7, (d) Hu-M10, (e) Hu-M11, and (f) Hu-M12. The banding patterns represent the allelic composition at the SSR locus (Section 2.2.2).

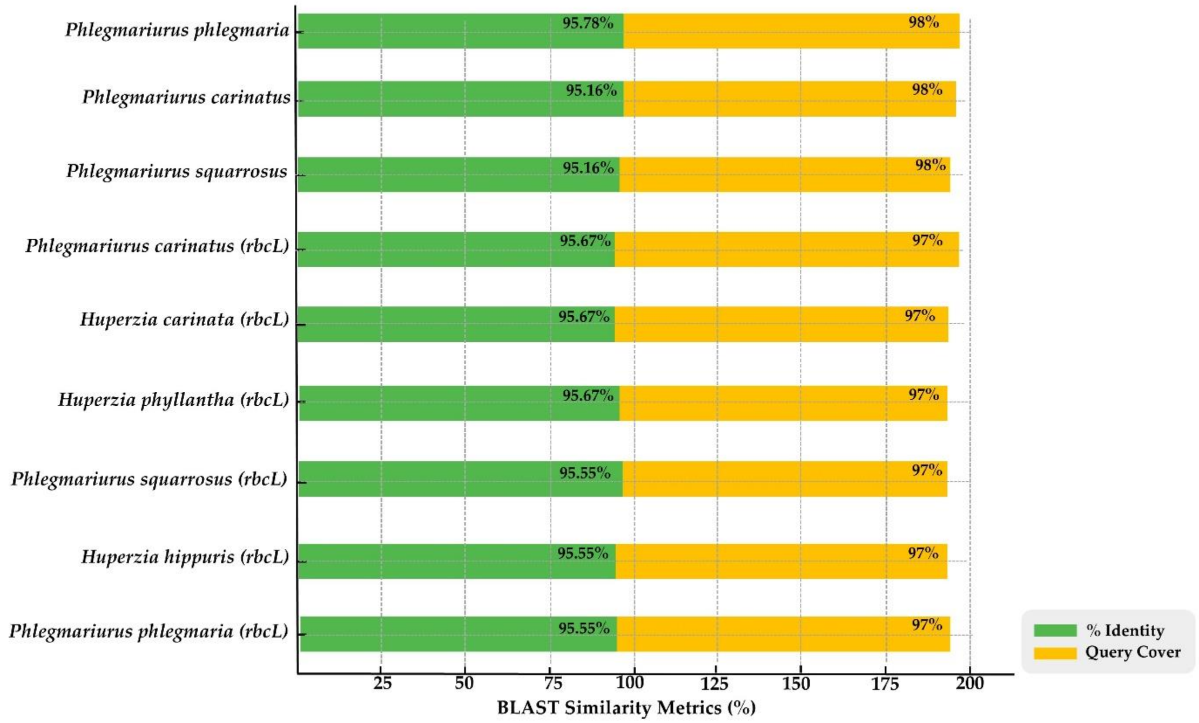

Supplementary Figure S3. BLAST alignment results showing 97.0–99.0% identity of *rbcL* sequences from 35 *Phlegmariurus* and one *Lycopodiella* accessions matching *P. phlegmaria*, *P. carinatus*, *P. squarrosus*, and *Huperzia phyllantha* compare to published reference sequences in the NCBI GenBank database (Section 2.3.1).

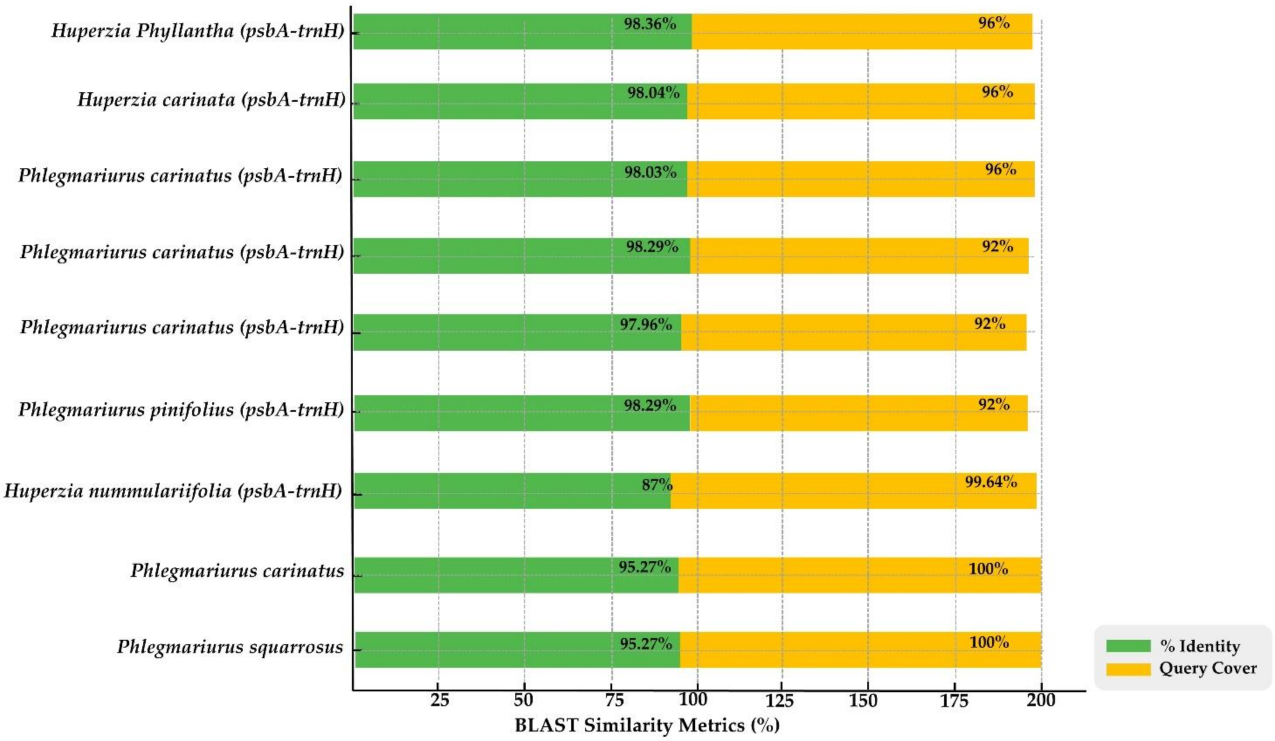

Supplementary Figure S4. BLAST similarity analysis of *psb*A-*trn*H sequences from 35 *Phlegmariurus* and one *Lycopodiella* compared to reference sequences from the NCBI database. The sequences matching *P. carinatus*, *P. squarrosus*, *Huperzia phyllantha*, and *P. pinifoli*us, with percentage identity (% Identity) ranging from 94.97% to 100%. Query cover ranged from 87% to 99%, and E-values were all highly significant (e-value < 1e-152) (Section 2.3.2).

Supplementary Table S1. Qualitative characters of fertile plant of *Phlegmariurus* sp. and *Lycopodiella* with their methods of scoring (Section 2.1.1).

No.	Sample	Leaf shape	Leaf angle to stem	Leaf apex	Leaf base	Leaf attachment
1	NST01	1	1	1	5	1
2	NST02	5	3	5	7	5
3	NST03	3	3	1	1	1
4	JP04	3	3	1	1	1
5	JP05	3	3	1	1	5
6	PNA06	3	3	1	1	1
7	NWT07	3	3	1	1	5
8	STN08	1	1	1	7	1
9	NST09	3	5	1	7	5
10	NST010	3	5	1	7	5
11	STN11	3	5	5	7	1
12	STN12	3	3	1	7	1
13	NST13	3	5	1	5	1
14	PNA14	3	1	1	7	1
15	NST15	7	1	1	5	1
16	PHI16	3	1	1	3	1
17	NST17	3	3	1	5	1
18	STN18	3	3	1	5	1
19	CPN19	3	1	1	5	1
20	NST20	1	3	1	5	1
21	NST21	3	3	1	1	1
22	NST22	3	3	1	5	1
23	NST23	1	1	1	5	1
24	JP24	No data	No data	No data	No data	No data
25	SKA25	3	3	1	5	1
26	JP24	3	3	1	5	1
27	KRB27	3	3	1	5	1
28	PNA28	3	3	1	7	5
29	PHI29	5	3	1	5	1
30	NST30	No data	No data	No data	No data	No data
31	MY31	3	3	1	5	1
32	MY32	3	3	1	5	1
33	MY33	3	3	1	5	1
34	NST34	3	3	1	5	1
35	LY35	3	3	1	5	1
36	NST36	3	3	1	5	1

Leaf shape; 1=ovate, 3= lanceolate, 5= ovate–lanceolate, 7= elliptic, Leaf angle of stem; 1=15°, 3=45°, 5=90°, Leaf apex; 1=acute, 5=mucronate, Leaf base; 1=acute, 3=attenuate, 5=obtuse, 7=truncate, Leaf attachment; 1=sessile, 5=subpetiolate.

Supplementary Table S2. Jaccard's genetic similarity coefficient matrix for 35 *Phlegmariurus* and one *Lycopodiella* accessions based on AFLP markers (Section 2.2.1).

sample	NST01	NST02	NST03	JP04	JP05	PNA06	NWT07	STW08	NST09	NST10	STN11	STN12	NST13	PNA14	NST15	PHI16	NST17	STN18	CPN19	NST20	NST21	NST22	NST23	JP24	SKA25	CPN26	KRB27	PNA28	PHI29	NST30	MY31	MY32	MY33	NST34	LYCO35	NST36
NST01	1.000	0.802	0.770	0.774	0.765	0.761	0.758	0.781	0.758	0.750	0.772	0.779	0.764	0.767	0.782	0.772	0.760	0.759	0.724	0.782	0.756	0.767	0.770	0.738	0.765	0.771	0.784	0.772	0.748	0.767	0.749	0.747	0.755	0.765	0.436	0.783
NST02	0.802	1.000	0.783	0.785	0.787	0.776	0.786	0.794	0.772	0.772	0.785	0.784	0.794	0.785	0.777	0.766	0.771	0.767	0.722	0.790	0.761	0.774	0.764	0.752	0.766	0.776	0.795	0.770	0.761	0.776	0.747	0.768	0.766	0.768	0.436	0.783
NST03	0.770	0.783	1.000	0.797	0.773	0.775	0.748	0.780	0.771	0.745	0.757	0.766	0.759	0.769	0.780	0.751	0.777	0.770	0.727	0.795	0.745	0.769	0.748	0.721	0.755	0.759	0.775	0.761	0.732	0.763	0.755	0.737	0.770	0.752	0.427	0.761
JP04	0.774	0.785	0.797	1.000	0.775	0.788	0.767	0.797	0.790	0.754	0.767	0.781	0.778	0.792	0.782	0.770	0.777	0.774	0.732	0.818	0.773	0.769	0.757	0.727	0.780	0.752	0.775	0.774	0.745	0.782	0.747	0.764	0.774	0.762	0.440	0.774
JP05	0.765	0.787	0.773	0.775	1.000	0.783	0.768	0.800	0.795	0.753	0.760	0.778	0.781	0.769	0.788	0.760	0.778	0.767	0.721	0.796	0.766	0.775	0.766	0.740	0.771	0.766	0.783	0.771	0.740	0.779	0.737	0.764	0.767	0.774	0.445	0.777
PNA06	0.761	0.776	0.775	0.788	0.783	1.000	0.772	0.790	0.791	0.762	0.771	0.778	0.771	0.766	0.792	0.761	0.786	0.765	0.721	0.794	0.759	0.777	0.756	0.750	0.773	0.766	0.787	0.762	0.755	0.771	0.742	0.768	0.777	0.766	0.432	0.767
NWT07	0.758	0.786	0.748	0.767	0.768	0.772	1.000	0.794	0.789	0.753	0.760	0.772	0.773	0.785	0.767	0.767	0.753	0.765	0.743	0.790	0.747	0.756	0.752	0.761	0.763	0.758	0.789	0.762	0.745	0.762	0.735	0.764	0.760	0.759	0.430	0.769
STW08	0.781	0.794	0.780	0.797	0.800	0.790	0.794	1.000	0.815	0.762	0.789	0.794	0.801	0.795	0.804	0.789	0.794	0.787	0.737	0.814	0.775	0.791	0.774	0.757	0.795	0.776	0.799	0.780	0.775	0.786	0.766	0.762	0.774	0.790	0.443	0.793
NST09	0.758	0.772	0.771	0.790	0.795	0.791	0.789	0.815	1.000	0.782	0.792	0.801	0.798	0.777	0.784	0.784	0.787	0.772	0.740	0.784	0.757	0.790	0.773	0.755	0.779	0.770	0.791	0.773	0.751	0.781	0.746	0.789	0.773	0.774	0.449	0.775
NST10	0.750	0.772	0.745	0.754	0.753	0.762	0.753	0.762	0.782	1.000	0.785	0.780	0.765	0.758	0.766	0.758	0.748	0.761	0.719	0.775	0.732	0.756	0.741	0.740	0.754	0.747	0.767	0.751	0.727	0.760	0.733	0.747	0.741	0.736	0.444	0.758
STN11	0.772	0.785	0.757	0.767	0.760	0.771	0.760	0.789	0.792	0.785	1.000	0.808	0.782	0.790	0.799	0.782	0.772	0.764	0.718	0.780	0.760	0.772	0.761	0.745	0.761	0.769	0.783	0.767	0.741	0.782	0.736	0.756	0.759	0.762	0.440	0.757
STN12	0.779	0.784	0.766	0.781	0.778	0.778	0.772	0.794	0.801	0.780	0.808	1.000	0.777	0.818	0.798	0.782	0.772	0.780	0.732	0.802	0.768	0.777	0.775	0.735	0.767	0.777	0.787	0.787	0.744	0.791	0.756	0.789	0.773	0.761	0.426	0.771
NST13	0.764	0.794	0.759	0.778	0.781	0.771	0.773	0.801	0.798	0.765	0.782	0.777	1.000	0.784	0.799	0.772	0.785	0.770	0.729	0.791	0.739	0.784	0.765	0.740	0.757	0.769	0.788	0.792	0.748	0.772	0.743	0.756	0.751	0.763	0.436	0.774
PNA14	0.767	0.785	0.769	0.792	0.769	0.766	0.785	0.795	0.777	0.758	0.790	0.818	0.784	1.000	0.797	0.789	0.772	0.782	0.734	0.797	0.762	0.778	0.760	0.748	0.769	0.773	0.789	0.775	0.753	0.771	0.748	0.764	0.769	0.764	0.440	0.767
NST15	0.782	0.777	0.780	0.782	0.788	0.792	0.767	0.804	0.784	0.766	0.799	0.798	0.799	0.797	1.000	0.791	0.785	0.798	0.745	0.818	0.775	0.774	0.780	0.768	0.791	0.792	0.800	0.780	0.758	0.782	0.751	0.764	0.761	0.777	0.453	0.780
PHI16	0.772	0.766	0.751	0.770	0.760	0.761	0.767	0.789	0.784	0.758	0.782	0.782	0.772	0.789	0.791	1.000	0.777	0.777	0.735	0.793	0.771	0.753	0.757	0.738	0.768	0.784	0.777	0.759	0.747	0.770	0.738	0.761	0.753	0.788	0.434	0.772
NST17	0.760	0.771	0.777	0.777	0.778	0.786	0.753	0.794	0.787	0.748	0.772	0.772	0.785	0.772	0.785	0.777	1.000	0.781	0.736	0.789	0.770	0.787	0.768	0.739	0.777	0.782	0.776	0.744	0.767	0.772	0.745	0.763	0.756	0.770	0.446	0.773
STN18	0.759	0.767	0.770	0.774	0.767	0.765	0.765	0.787	0.772	0.761	0.764	0.780	0.770	0.782	0.798	0.777	0.781	1.000	0.744	0.810	0.771	0.764	0.766	0.743	0.768	0.765	0.765	0.761	0.746	0.776	0.749	0.761	0.753	0.769	0.434	0.768
CPN19	0.724	0.722	0.727	0.732	0.721	0.721	0.743	0.737	0.740	0.719	0.718	0.732	0.729	0.734	0.745	0.735	0.736	0.744	1.000	0.773	0.732	0.717	0.718	0.725	0.737	0.721	0.742	0.722	0.700	0.756	0.701	0.736	0.731	0.736	0.419	0.743
NST20	0.782	0.790	0.795	0.818	0.796	0.794	0.790	0.814	0.784	0.775	0.780	0.802	0.791	0.797	0.818	0.793	0.789	0.810	0.773	1.000	0.792	0.797	0.780	0.759	0.778	0.790	0.801	0.775	0.755	0.805	0.757	0.787	0.780	0.785	0.448	0.793
NST21	0.756	0.761	0.745	0.773	0.766	0.759	0.747	0.775	0.757	0.732	0.760	0.768	0.739	0.762	0.775	0.771	0.770	0.771	0.732	0.792	1.000	0.764	0.771	0.718	0.747	0.760	0.772	0.747	0.744	0.773	0.740	0.751	0.750	0.749	0.423	0.773
NST22	0.767	0.774	0.769	0.769	0.775	0.777	0.756	0.791	0.790	0.756	0.772	0.777	0.784	0.778	0.774	0.753	0.787	0.764	0.717	0.797	0.764	1.000	0.782	0.744	0.767	0.775	0.796	0.769	0.756	0.778	0.751	0.771	0.761	0.764	0.432	0.768
NST23	0.770	0.764	0.748	0.757	0.766	0.756	0.752	0.774	0.773	0.741	0.761	0.775	0.765	0.760	0.780	0.757	0.768	0.766	0.718	0.780	0.771	0.782	1.000	0.730	0.761	0.758	0.791	0.759	0.743	0.765	0.747	0.762	0.765	0.767	0.431	0.765
JP24	0.738	0.752	0.721	0.727	0.740	0.750	0.761	0.757	0.755	0.740	0.745	0.735	0.740	0.748	0.768	0.738	0.739	0.743	0.725	0.759	0.718	0.744	0.730	1.000	0.751	0.744	0.758	0.747	0.722	0.748	0.737	0.750	0.726	0.759	0.442	0.760
SKA25	0.765	0.766	0.755	0.780	0.771	0.773	0.763	0.795	0.779	0.754	0.761	0.767	0.757	0.769	0.791	0.768	0.777	0.768	0.737	0.778	0.747	0.767	0.761	0.751	1.000	0.779	0.783	0.780	0.760	0.782	0.743	0.762	0.776	0.788	0.443	0.765
CPN26	0.771	0.776	0.759	0.752	0.766	0.766	0.758	0.776	0.770	0.747	0.769	0.777	0.769	0.773	0.792	0.784	0.782	0.765	0.721	0.790	0.760	0.775	0.758	0.744	0.779	1.000	0.818	0.794	0.770	0.788	0.744	0.760	0.754	0.770	0.419	0.771
KRB27	0.784	0.795	0.775	0.775	0.783	0.787	0.789	0.799	0.791	0.767	0.783	0.787	0.788	0.789	0.800	0.777	0.776	0.765	0.742	0.801	0.772	0.796	0.791	0.758	0.783	0.818	1.000	0.810	0.774	0.790	0.782	0.793	0.775	0.795	0.436	0.803
PNA28	0.772	0.770	0.761	0.774	0.771	0.762	0.762	0.780	0.773	0.751	0.767	0.787	0.792	0.775	0.780	0.759	0.744	0.761	0.722	0.775	0.747	0.769	0.759	0.747	0.780	0.794	0.810	1.000	0.764	0.794	0.764	0.769	0.776	0.769	0.424	0.782
PHI29	0.748	0.761	0.732	0.745	0.740	0.755	0.745	0.775	0.751	0.727	0.741	0.744	0.748	0.753	0.758	0.747	0.767	0.746	0.700	0.755	0.744	0.756	0.743	0.722	0.760	0.770	0.774	0.764	1.000	0.764	0.755	0.753	0.760	0.768	0.429	0.756
NST30	0.767	0.776	0.763	0.782	0.779	0.771	0.762	0.786	0.781	0.760	0.782	0.791	0.772	0.771	0.782	0.770	0.772	0.776	0.756	0.805	0.773	0.778	0.765	0.748	0.782	0.788	0.790	0.794	0.764	1.000	0.766	0.791	0.778	0.773	0.440	0.784
MY31	0.749	0.747	0.755	0.747	0.737	0.742	0.735	0.766	0.746	0.733	0.736	0.756	0.743	0.748	0.751	0.738	0.745	0.749	0.701	0.757	0.740	0.751	0.747	0.737	0.743	0.744	0.782	0.764	0.755	0.766	1.000	0.757	0.772	0.754	0.410	0.774
MY32	0.747	0.768	0.737	0.764	0.764	0.768	0.764	0.762	0.789	0.747	0.756	0.789	0.756	0.764	0.764	0.761	0.763	0.761	0.736	0.787	0.751	0.771	0.762	0.750	0.762	0.760	0.793	0.769	0.753	0.791	0.757	1.000	0.767	0.787	0.447	0.773
MY33	0.755	0.766	0.770	0.774	0.767	0.777	0.760	0.774	0.773	0.741	0.759	0.773	0.751	0.769	0.761	0.753	0.756	0.753	0.731	0.780	0.750	0.761	0.765	0.726	0.776	0.754	0.775	0.776	0.760	0.778	0.772	0.767	1.000	0.775	0.438	0.772
NST34	0.765	0.768	0.752	0.762	0.774	0.766	0.759	0.790	0.774	0.736	0.762	0.761	0.763	0.764	0.777	0.788	0.770	0.769	0.736	0.785	0.749	0.764	0.767	0.759	0.788	0.770	0.795	0.769	0.768	0.773	0.754	0.787	0.775	1.000	0.437	0.788
LYCO35	0.436	0.436	0.427	0.440	0.445	0.432	0.430	0.443	0.449	0.444	0.440	0.426	0.436	0.440	0.453	0.434	0.446	0.434	0.419	0.448	0.423	0.432	0.431	0.442	0.443	0.419	0.436	0.424	0.429	0.440	0.410	0.447	0.438	0.437	1.000	0.455
NST36	0.783	0.783	0.761	0.774	0.777	0.767	0.769	0.793	0.775	0.758	0.757	0.771	0.774	0.767	0.780	0.772	0.773	0.768	0.743	0.793	0.773	0.768	0.765	0.760	0.765	0.771	0.803	0.782	0.756	0.784	0.774	0.773	0.772	0.788	0.455	1.000

Supplementary Table S3. Jaccard's genetic similarity coefficient matrix for 35 *Phlegmariurus* and one *Lycopodiella* accessions based on SSR markers (Section 2.2.2).

sample	NST01	NST02	NST03	JP04	JP05	PNA06	NWT07	STN08	NST09	NST10	STN11	STN12	NST13	PNA14	NST15	PHI16	NST17	STN18
**NST01**	1.000	0.778	0.333	0.778	0.308	0.417	0.455	0.545	0.545	0.308	0.333	0.308	0.333	0.333	0.385	0.333	0.286	0.308
**NST02**	0.778	1.000	0.231	0.600	0.214	0.308	0.333	0.417	0.417	0.214	0.231	0.308	0.231	0.231	0.286	0.231	0.200	0.214
**NST03**	0.333	0.231	1.000	0.455	0.308	0.308	0.333	0.417	0.308	0.308	0.333	0.214	0.333	0.455	0.200	0.455	0.636	0.545
**JP04**	0.778	0.600	0.455	1.000	0.308	0.417	0.455	0.545	0.545	0.308	0.333	0.308	0.333	0.333	0.385	0.333	0.286	0.308
**JP05**	0.308	0.214	0.308	0.308	1.000	0.636	0.545	0.500	0.500	0.500	0.417	0.385	0.545	0.308	0.357	0.308	0.357	0.500
**PNA06**	0.417	0.308	0.308	0.417	0.636	1.000	0.545	0.636	0.636	0.500	0.545	0.500	0.417	0.308	0.462	0.308	0.357	0.385
**NWT07**	0.455	0.333	0.333	0.455	0.545	0.545	1.000	0.417	0.417	0.417	0.455	0.417	0.455	0.333	0.286	0.333	0.286	0.417
**STN08**	0.545	0.417	0.417	0.545	0.500	0.636	0.417	1.000	0.800	0.385	0.308	0.385	0.308	0.308	0.583	0.308	0.462	0.385
**NST09**	0.545	0.417	0.308	0.545	0.500	0.636	0.417	0.800	1.000	0.500	0.308	0.385	0.308	0.308	0.583	0.308	0.357	0.385
**NST10**	0.308	0.214	0.308	0.308	0.500	0.500	0.417	0.385	0.500	1.000	0.700	0.500	0.545	0.308	0.267	0.308	0.357	0.385
**STN11**	0.333	0.231	0.333	0.333	0.417	0.545	0.455	0.308	0.308	0.700	1.000	0.417	0.600	0.333	0.200	0.333	0.286	0.308
**STN12**	0.308	0.308	0.214	0.308	0.385	0.500	0.417	0.385	0.385	0.500	0.417	1.000	0.417	0.214	0.267	0.308	0.267	0.286
**NST13**	0.333	0.231	0.333	0.333	0.545	0.417	0.455	0.308	0.308	0.545	0.600	0.417	1.000	0.455	0.286	0.333	0.286	0.308
**PNA14**	0.333	0.231	0.455	0.333	0.308	0.308	0.333	0.308	0.308	0.308	0.333	0.214	0.455	1.000	0.286	0.778	0.385	0.545
**NST15**	0.385	0.286	0.200	0.385	0.357	0.462	0.286	0.583	0.583	0.267	0.200	0.267	0.286	0.286	1.000	0.200	0.250	0.267
**PHI16**	0.333	0.231	0.455	0.333	0.308	0.308	0.333	0.308	0.308	0.308	0.333	0.308	0.333	0.778	0.200	1.000	0.385	0.545
**NST17**	0.286	0.200	0.636	0.286	0.357	0.357	0.286	0.462	0.357	0.357	0.286	0.267	0.286	0.385	0.250	0.385	1.000	0.583
**STN18**	0.308	0.214	0.545	0.308	0.500	0.385	0.417	0.385	0.385	0.385	0.308	0.286	0.308	0.545	0.267	0.545	0.583	1.000
**CPN19**	0.333	0.231	0.455	0.333	0.417	0.545	0.455	0.308	0.308	0.417	0.600	0.308	0.455	0.333	0.200	0.333	0.385	0.417
**NST20**	0.455	0.333	0.333	0.455	0.417	0.545	0.778	0.417	0.417	0.417	0.455	0.545	0.455	0.333	0.286	0.455	0.286	0.308
**NST21**	0.385	0.286	0.385	0.385	0.462	0.462	0.500	0.462	0.462	0.357	0.286	0.357	0.286	0.500	0.333	0.500	0.538	0.727
**NST22**	0.333	0.231	0.333	0.333	0.417	0.545	0.600	0.308	0.308	0.417	0.600	0.308	0.455	0.455	0.200	0.455	0.286	0.308
**NST23**	0.417	0.308	0.308	0.308	0.800	0.500	0.545	0.385	0.385	0.500	0.417	0.385	0.545	0.308	0.267	0.308	0.357	0.500
**JP24**	0.333	0.231	0.333	0.333	0.214	0.308	0.455	0.417	0.308	0.214	0.231	0.214	0.231	0.231	0.286	0.231	0.286	0.214
**SKA25**	0.308	0.214	0.214	0.308	0.385	0.500	0.417	0.385	0.385	0.385	0.308	0.385	0.417	0.308	0.583	0.214	0.267	0.286
**CPN26**	0.455	0.333	0.333	0.455	0.417	0.545	0.600	0.417	0.417	0.545	0.600	0.700	0.600	0.333	0.286	0.455	0.286	0.308
**KRB27**	0.417	0.308	0.308	0.417	0.385	0.500	0.545	0.385	0.385	0.500	0.545	0.500	0.700	0.417	0.357	0.308	0.357	0.286
**PNA28**	0.333	0.231	0.333	0.333	0.417	0.417	0.455	0.308	0.308	0.545	0.600	0.417	0.778	0.600	0.286	0.455	0.286	0.308
**PHI29**	0.308	0.214	0.308	0.308	0.500	0.500	0.417	0.385	0.385	0.500	0.417	0.385	0.545	0.417	0.357	0.308	0.357	0.385
**NST30**	0.417	0.308	0.417	0.417	0.500	0.636	0.417	0.636	0.636	0.385	0.308	0.385	0.417	0.545	0.583	0.417	0.462	0.500
**MY31**	0.214	0.133	0.214	0.214	0.286	0.286	0.214	0.286	0.286	0.385	0.308	0.200	0.308	0.308	0.357	0.214	0.267	0.286
**MY32**	0.333	0.231	0.333	0.333	0.545	0.417	0.600	0.308	0.308	0.545	0.600	0.308	0.455	0.333	0.200	0.333	0.286	0.417
**MY33**	0.385	0.286	0.286	0.385	0.357	0.462	0.500	0.357	0.357	0.357	0.385	0.357	0.500	0.385	0.333	0.286	0.333	0.267
**NST34**	0.417	0.308	0.308	0.417	0.385	0.500	0.545	0.385	0.385	0.385	0.417	0.500	0.417	0.308	0.267	0.417	0.267	0.286
**LYCO35**	0.125	0.059	0.059	0.125	0.118	0.188	0.200	0.118	0.118	0.118	0.125	0.118	0.125	0.059	0.176	0.059	0.053	0.056
**NST36**	0.385	0.286	0.385	0.385	0.462	0.583	0.500	0.583	0.462	0.462	0.385	0.462	0.385	0.286	0.429	0.286	0.429	0.357

sample	CPN19	NST20	NST21	NST22	NST23	JP24	SKA25	CPN26	KRB27	PNA28	PHI29	NST30	MY31	MY32	MY33	NST34	LYCO35	NST36
**NST01**	0.333	0.455	0.385	0.333	0.417	0.333	0.308	0.455	0.417	0.333	0.308	0.417	0.214	0.333	0.385	0.417	0.125	0.385
**NST02**	0.231	0.333	0.286	0.231	0.308	0.231	0.214	0.333	0.308	0.231	0.214	0.308	0.133	0.231	0.286	0.308	0.059	0.286
**NST03**	0.455	0.333	0.385	0.333	0.308	0.333	0.214	0.333	0.308	0.333	0.308	0.417	0.214	0.333	0.286	0.308	0.059	0.385
**JP04**	0.333	0.455	0.385	0.333	0.308	0.333	0.308	0.455	0.417	0.333	0.308	0.417	0.214	0.333	0.385	0.417	0.125	0.385
**JP05**	0.417	0.417	0.462	0.417	0.800	0.214	0.385	0.417	0.385	0.417	0.500	0.500	0.286	0.545	0.357	0.385	0.118	0.462
**PNA06**	0.545	0.545	0.462	0.545	0.500	0.308	0.500	0.545	0.500	0.417	0.500	0.636	0.286	0.417	0.462	0.500	0.188	0.583
**NWT07**	0.455	0.778	0.500	0.600	0.545	0.455	0.417	0.600	0.545	0.455	0.417	0.417	0.214	0.600	0.500	0.545	0.200	0.500
**STN08**	0.308	0.417	0.462	0.308	0.385	0.417	0.385	0.417	0.385	0.308	0.385	0.636	0.286	0.308	0.357	0.385	0.118	0.583
**NST09**	0.308	0.417	0.462	0.308	0.385	0.308	0.385	0.417	0.385	0.308	0.385	0.636	0.286	0.308	0.357	0.385	0.118	0.462
**NST10**	0.417	0.417	0.357	0.417	0.500	0.214	0.385	0.545	0.500	0.545	0.500	0.385	0.385	0.545	0.357	0.385	0.118	0.462
**STN11**	0.600	0.455	0.286	0.600	0.417	0.231	0.308	0.600	0.545	0.600	0.417	0.308	0.308	0.600	0.385	0.417	0.125	0.385
**STN12**	0.308	0.545	0.357	0.308	0.385	0.214	0.385	0.700	0.500	0.417	0.385	0.385	0.200	0.308	0.357	0.500	0.118	0.462
**NST13**	0.455	0.455	0.286	0.455	0.545	0.231	0.417	0.600	0.700	0.778	0.545	0.417	0.308	0.455	0.500	0.417	0.125	0.385
**PNA14**	0.333	0.333	0.500	0.455	0.308	0.231	0.308	0.333	0.417	0.600	0.417	0.545	0.308	0.333	0.385	0.308	0.059	0.286
**NST15**	0.200	0.286	0.333	0.200	0.267	0.286	0.583	0.286	0.357	0.286	0.357	0.583	0.357	0.200	0.333	0.267	0.176	0.429
**PHI16**	0.333	0.455	0.500	0.455	0.308	0.231	0.214	0.455	0.308	0.455	0.308	0.417	0.214	0.333	0.286	0.417	0.059	0.286
**NST17**	0.385	0.286	0.538	0.286	0.357	0.286	0.267	0.286	0.357	0.286	0.357	0.462	0.267	0.286	0.333	0.267	0.053	0.429
**STN18**	0.417	0.308	0.727	0.308	0.500	0.214	0.286	0.308	0.286	0.308	0.385	0.500	0.286	0.417	0.267	0.286	0.056	0.357
**CPN19**	1.000	0.455	0.286	0.600	0.417	0.231	0.417	0.455	0.417	0.455	0.545	0.308	0.308	0.600	0.500	0.545	0.200	0.500
**NST20**	0.455	1.000	0.385	0.600	0.417	0.455	0.417	0.778	0.545	0.455	0.417	0.417	0.214	0.455	0.500	0.700	0.200	0.500
**NST21**	0.286	0.385	1.000	0.286	0.462	0.286	0.357	0.385	0.462	0.286	0.357	0.583	0.267	0.385	0.429	0.357	0.111	0.429
**NST22**	0.600	0.600	0.286	1.000	0.417	0.333	0.308	0.455	0.417	0.600	0.417	0.308	0.214	0.455	0.385	0.417	0.125	0.385
**NST23**	0.417	0.417	0.462	0.417	1.000	0.214	0.385	0.417	0.385	0.417	0.500	0.385	0.286	0.545	0.357	0.385	0.118	0.462
**JP24**	0.231	0.455	0.286	0.333	0.214	1.000	0.308	0.333	0.308	0.231	0.214	0.308	0.417	0.231	0.286	0.308	0.125	0.385
**SKA25**	0.417	0.417	0.357	0.308	0.385	0.308	1.000	0.417	0.500	0.417	0.636	0.500	0.500	0.417	0.583	0.500	0.267	0.583
**CPN26**	0.455	0.778	0.385	0.455	0.417	0.333	0.417	1.000	0.700	0.600	0.417	0.417	0.214	0.455	0.500	0.700	0.200	0.500
**KRB27**	0.417	0.545	0.462	0.417	0.385	0.308	0.500	0.700	1.000	0.700	0.500	0.500	0.286	0.417	0.727	0.500	0.188	0.462
**PNA28**	0.455	0.455	0.286	0.600	0.417	0.231	0.417	0.600	0.700	1.000	0.545	0.417	0.308	0.455	0.500	0.417	0.125	0.385
**PHI29**	0.545	0.417	0.357	0.417	0.500	0.214	0.636	0.417	0.500	0.545	1.000	0.500	0.500	0.545	0.583	0.500	0.188	0.583
**NST30**	0.308	0.417	0.583	0.308	0.385	0.308	0.500	0.417	0.500	0.417	0.500	1.000	0.385	0.308	0.462	0.385	0.118	0.462
**MY31**	0.308	0.214	0.267	0.214	0.286	0.417	0.500	0.214	0.286	0.308	0.500	0.385	1.000	0.417	0.357	0.286	0.118	0.357
**MY32**	0.600	0.455	0.385	0.455	0.545	0.231	0.417	0.455	0.417	0.455	0.545	0.308	0.417	1.000	0.500	0.545	0.200	0.500
**MY33**	0.500	0.500	0.429	0.385	0.357	0.286	0.583	0.500	0.727	0.500	0.583	0.462	0.357	0.500	1.000	0.583	0.250	0.538
**NST34**	0.545	0.700	0.357	0.417	0.385	0.308	0.500	0.700	0.500	0.417	0.500	0.385	0.286	0.545	0.583	1.000	0.267	0.583
**LYCO35**	0.200	0.200	0.111	0.125	0.118	0.125	0.267	0.200	0.188	0.125	0.188	0.118	0.118	0.200	0.250	0.267	1.000	0.333
**NST36**	0.500	0.500	0.429	0.385	0.462	0.385	0.583	0.500	0.462	0.385	0.583	0.462	0.357	0.500	0.538	0.583	0.333	1.000

Supplementary Table S4. GenBase accession number of *Phlegmariurus* specimens and one *Lycopodiella* as outgroup used in this study (Section 2.3.1, 2.3.2, 4.1).

No.	Common name	Sample ID	Collection site	*rbc*L Accession No.	*psb*A-*trn*H Accession No.
1	Raya Kled Hoi	NST01	Nakhon Si Thammarat, Thailand	C_AA107161.1	C_AA107125.1
2	Hang Hong	NST02	Nakhon Si Thammarat, Thailand	C_AA107162.1	C_AA107126.1
3	Soi Samliem	NST03	Nakhon Si Thammarat, Thailand	C_AA107163.1	C_AA107127.1
4	Chong Blue	JP04	Japan	C_AA107153.1	C_AA107117.1
5	Chong Blue Klai	JP05	Japan	C_AA107154.1	C_AA107118.1
6	Hang Sing Klai Pang–nga	PNA06	Pang–nga, Thailand	C_AA107179.1	C_AA107143.1
7	Nang Klai Sukirin	NWT07	Narathiwat, Thailand	C_AA107176.1	C_AA107140.1
8	Sam Lium Satun	STN08	Satun, Thailand	C_AA107183.1	C_AA107147.1
9	Chong Krae Khao Luang	NST09	Nakhon Si Thammarat, Thailand	C_AA107164.1	C_AA107128.1
10	Chong Blue Kiew	NST10	Nakhon Si Thammarat, Thailand	C_AA107165.1	C_AA107129.1
11	Chong Baisorn Satun	STN11	Satun, Thailand	C_AA107184.1	C_AA107148.1
12	Soi Nangklai Satun	STN12	Satun, Thailand	C_AA107185.1	C_AA107149.1
13	Chong Nangklee Kankaw	NST13	Nakhon Si Thammarat, Thailand	C_AA107166.1	C_AA107130.1
14	Soi Hoklium Pang–nga	PNA14	Pang–nga, Thailand	C_AA107180.1	C_AA107144.1
15	Kled Hoi Silium	NST15	Nakhon Si Thammarat, Thailand	C_AA107167.1	C_AA107131.1
16	Sing Philippines	PHI16	Philippines	C_AA107177.1	C_AA107141.1
17	Soi Anyamanee	NST17	Nakhon Si Thammarat, Thailand	C_AA107168.1	C_AA107132.1
18	Sing Klai Satun	STN18	Satun, Thailand	C_AA107186.1	C_AA107150.1
19	Soi Naree Chumphon	CPN19	Chumphon, Thailand	C_AA107151.1	C_AA107115.1
20	Chong Nakarat	NST20	Nakhon Si Thammarat, Thailand	C_AA107169.1	C_AA107133.1
21	Hang Sing	NST21	Nakhon Si Thammarat, Thailand	C_AA107170.1	C_AA107134.1
22	Hang Sing Soi	NST22	Nakhon Si Thammarat, Thailand	C_AA107171.1	C_AA107135.1
23	Soi Pakarang	NST23	Nakhon Si Thammarat, Thailand	C_AA107172.1	C_AA107136.1
24	Chong Mee	JP24	Japan	C_AA107155.1	C_AA107119.1
25	Soi Nangklay Songkhla	SKA25	Songkhla, Thailand	C_AA107182.1	C_AA107146.1
26	Soi Nangklay Chumpon	CPN26	Chumphon, Thailand	C_AA107152.1	C_AA107116.1
27	Soi Nangklay Krabi	KRB27	Krabi, Thailand	C_AA107156.1	C_AA107120.1

Supplementary Table S2. GenBase accession number of *Phlegmariurus* specimens and one *Lycopodiella* as outgroup used in this study (Section 2.3.1, 2.3.2, 4.1).

No.	Common name	Sample ID	Collection site	*rbc*L Accession No.	*psb*A-*trn*H Accession No.
28	Soi Nangklay Pang–gna	PNA28	Pang–nga, Thailand	C_AA107181.1	C_AA107145.1
29	Chong Krae Philippines	PHI29	Philippines	C_AA107178.1	C_AA107142.1
30	Soi Nang Krong Lium	NST30	Nakhon Si Thammarat, Thailand	C_AA107173.1	C_AA107137.1
31	Hang Sing Blue	MY31	Malaysia	C_AA107158.1	C_AA107122.1
32	Soi Solomon	MY32	The Solomon	C_AA107159.1	C_AA107123.1
33	Soi Carinata lium	MY33	Malaysia	C_AA107160.1	C_AA107124.1
34	Hang Noo Tawanauk	NST34	Nakhon Si Thammarat, Thailand	C_AA107174.1	C_AA107138.1
35	Samroiyod	LYCO35	Nakhon Si Thammarat, Thailand	C_AA107157.1	C_AA107121.1
36	Kled Hoi Hybrid	NST36	Nakhon Si Thammarat, Thailand	C_AA107175.1	C_AA107139.1

Supplementary Table S5. Primers and adapters sequences used for AFLP analysis (Section 4.3.2).

Primer pairs	Name/Abbreviation[1]	Type	Sequence (5'→3')
	*Eco*RI-adapter	Adapter +	CTCGTAGACTGCGTACC
		Adapter –	AATTGGTACGCAGTCTAC
	*Mse*I-adapter	Adapter +	GACGATGAGTCCTGAG
		Adapter –	TACTCAGGATCAT
	E+A	Primer +1	GACTGCGTACCAATTC**A**
1	ER-AA	Primer +2	GACTGCGTACCAATTC**AA**
2	ER-AC	Primer +2	GACTGCGTACCAATTC**AC**
3	ER-AG	Primer +2	GACTGCGTACCAATTC**AG**
4	ER-AT	Primer +2	GACTGCGTACCAATTC**AT**
5	ER-AAG	Primer +3	GACTGCGTACCAATTC**AAG**
6	ER-ACC	Primer +3	GACTGCGTACCAATTC**ACC**
7	ER-AGA	Primer +3	GACTGCGTACCAATTC**AGA**
8	ER-AGC	Primer +3	GACTGCGTACCAATTC**AGC**
	M+C	Primer +1	GATGAGTCCTGAGTAA**C**
1	MS-CAG	Primer +3	GATGAGTCCTGAGTAA**CAG**
2	MS-CCA	Primer +3	GATGAGTCCTGAGTAA**CCA**
3	MS-CGA	Primer +3	GATGAGTCCTGAGTAA**CGA**
4	MS-CGT	Primer +3	GATGAGTCCTGAGTAA**CGT**
5	MS-CTA	Primer +3	GATGAGTCCTGAGTAA**CTA**

[1]E = *Eco*RI and M = *Mse*I are bases of the selective nucleotide (s) added at 3' end.

Supplementary Table S6. SSR primers used for polymorphism analysis (Section 4.3.3).

Oligo name	Primer sequence (5'→3')	Annealing temperature (ºC)	Repeat motif
Hu-C1	F: GCATGTTGGGTTCTCAAAGCG<br>R: GGAAATTGGAGAAGCTACCGG	55	(TTTCTT)3
Hu-C2	F: CATGAGTATACTCCTTGGG<br>R: CTAGCTGTTTGTACGTAAAGG	55	(AT)14
Hu-C3	F: CTGAAGGATAACCGGATCC<br>R: GATTGGATTCCCAACATTTC	53	(AGATAT)3
Hu-C4	F: TACCCGGGTTGTCTCGACA<br>R: CCAGGAACTTCGATTGCACC	60	(TAAA)28
Hu-M5	F: CTATTTCGGCCTATTTGAGGTG<br>R: CCTATGTTTGAGGATGTGACGA	55	(AT)10
Hu-M6	F: GTCTAGTTCGTCGTATCTCCCG<br>R: TTATGTTCCCTCTTCTTGTGGC	60	(ATATCT)3
Hu-M7	F: GTCTAGTTCGTCGTATCTCCCG<br>R: CTATATCAGCGTCAATCACCC	62	(ATACTA)3
Hu-M8	F: TTACTCTCGGCCTCTGTGGTAT<br>R: CTCATTACCCTTAGCCAGCTTC	62	(ATT)6
Hu-M9	F: GTGCAAACAAAGCCATTTTACC<br>R: CTCTTCTTCAGCAGCCCATAAC	65	(AT)14
Hu-M10	F: AGCAAATCGTACCGGACTAGAA<br>R: GACGGATAAAGTATTGAACCTTGG	60	(AGATAT)3
Hu-M11	F: CCTATGTTTGAGGATGTGAGA<br>R: CTATTTCGGCCTATTTGAGGTG	53	(TAAA)28
Hu-M12	F: AGAAACGGAATCTCTTGTGGA<br>R: GATGGCTATGGGTGCTTTATTC	55	(AT)10
Hu-M13	F: TGTTTGATAAAGGGGAGCCTAAA<br>R: TAGAAAGGGAAGAAGCTCATGC	60	(TAT)8
